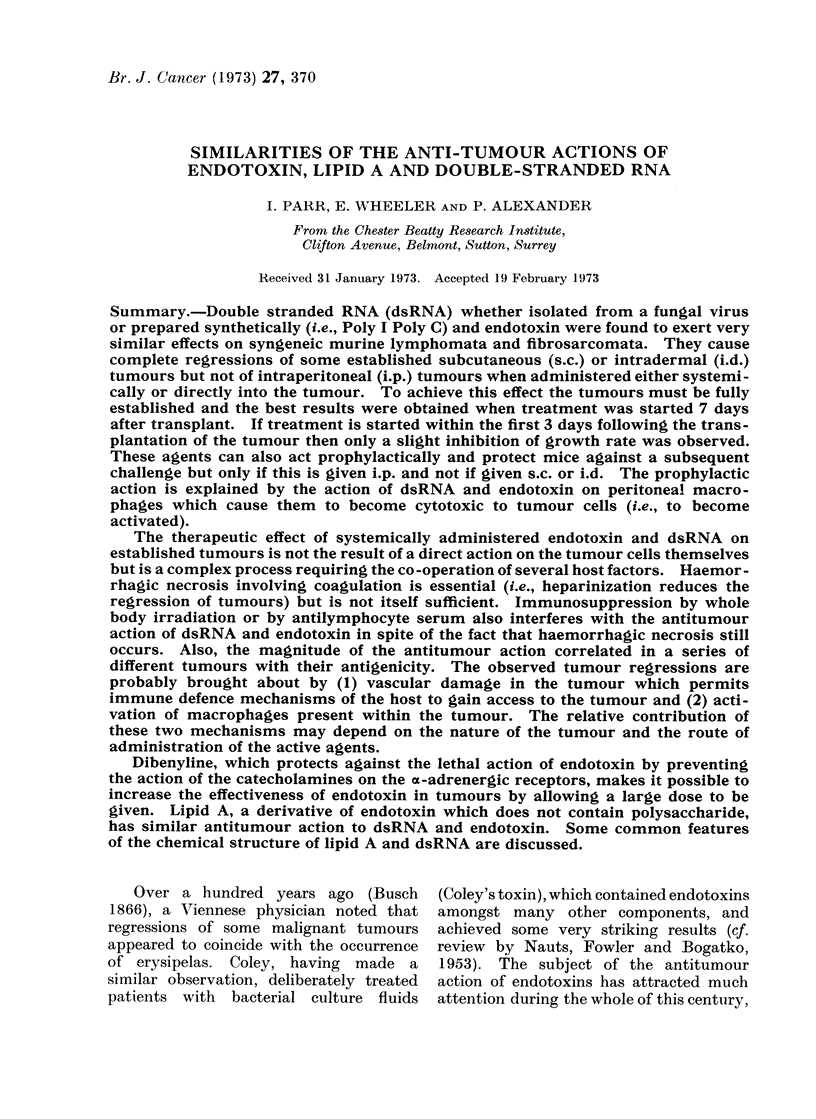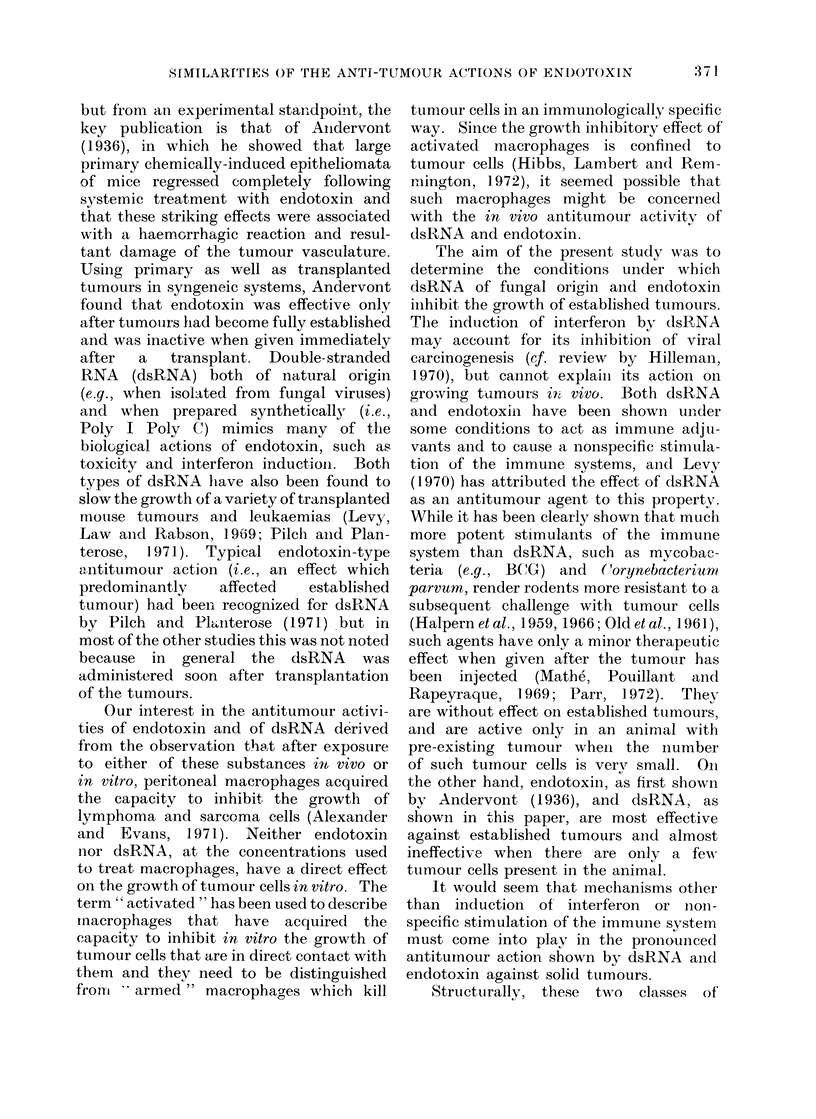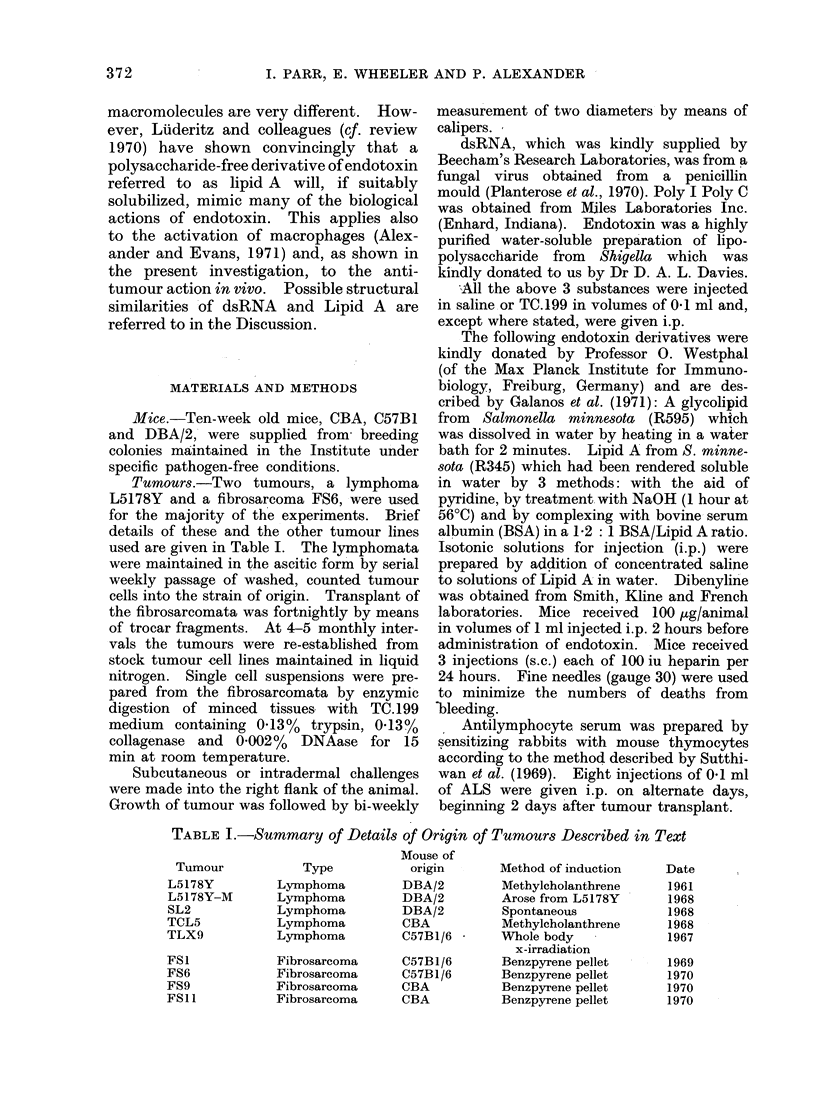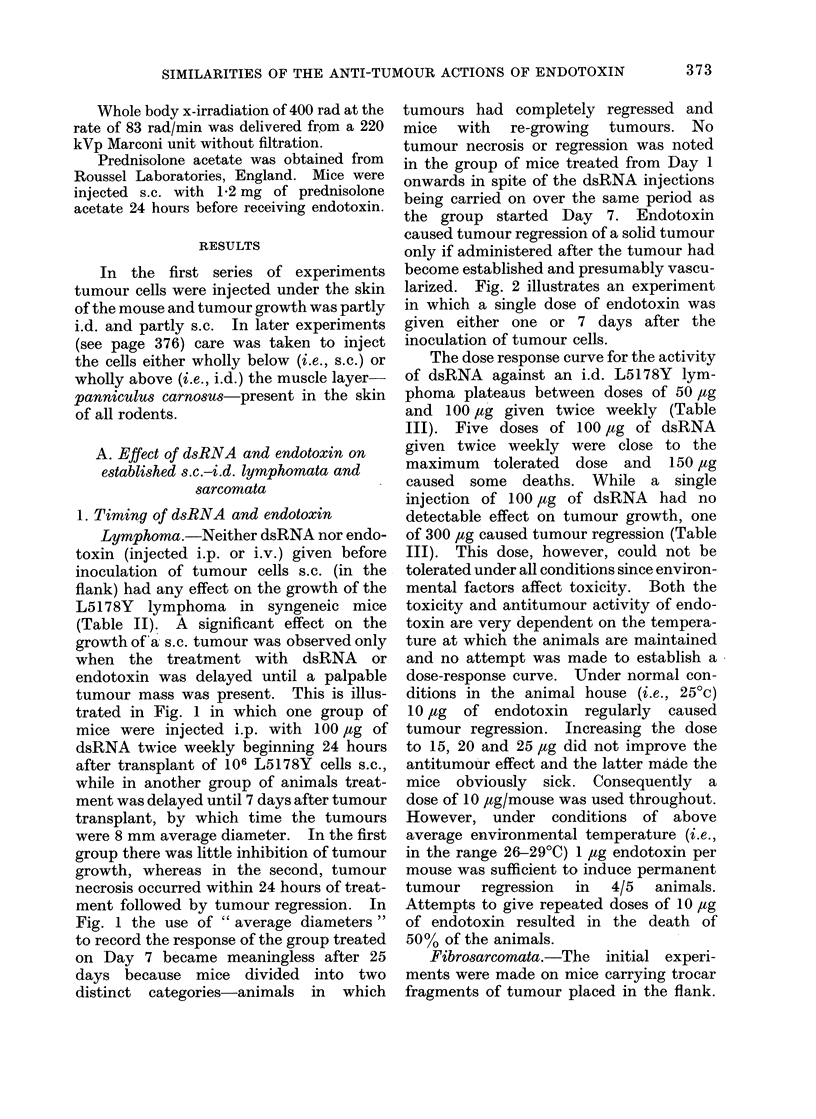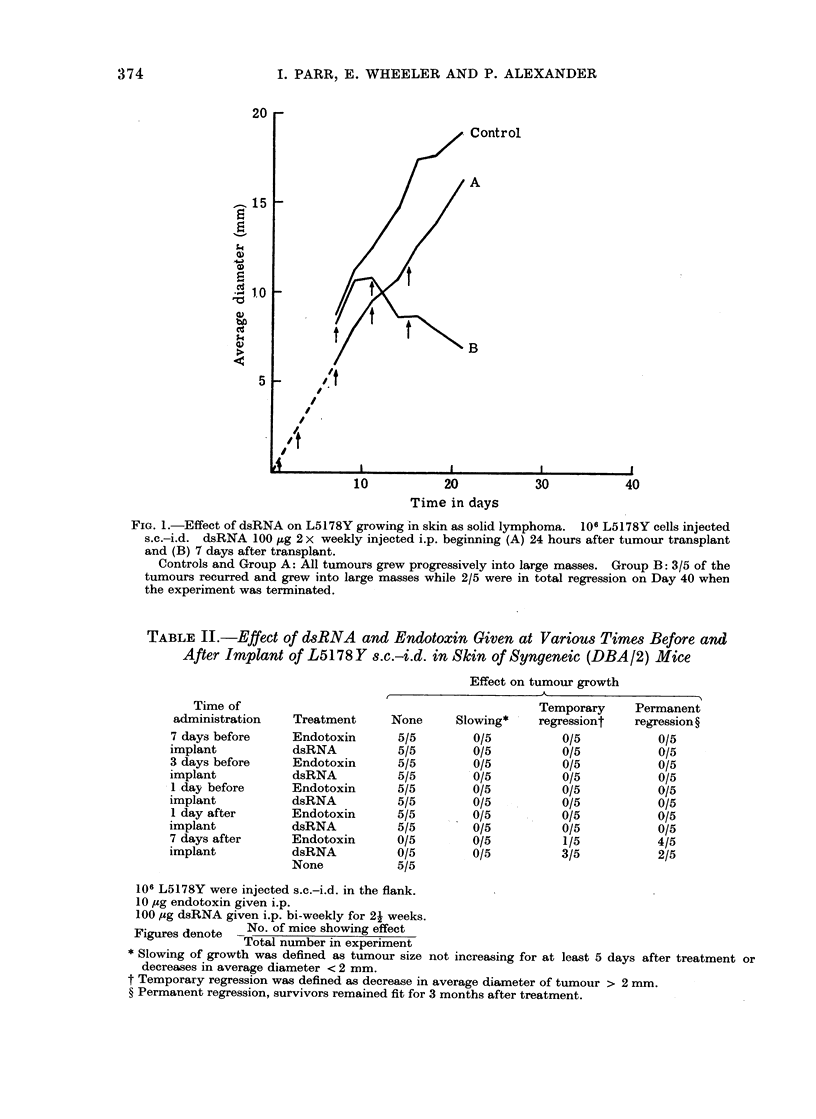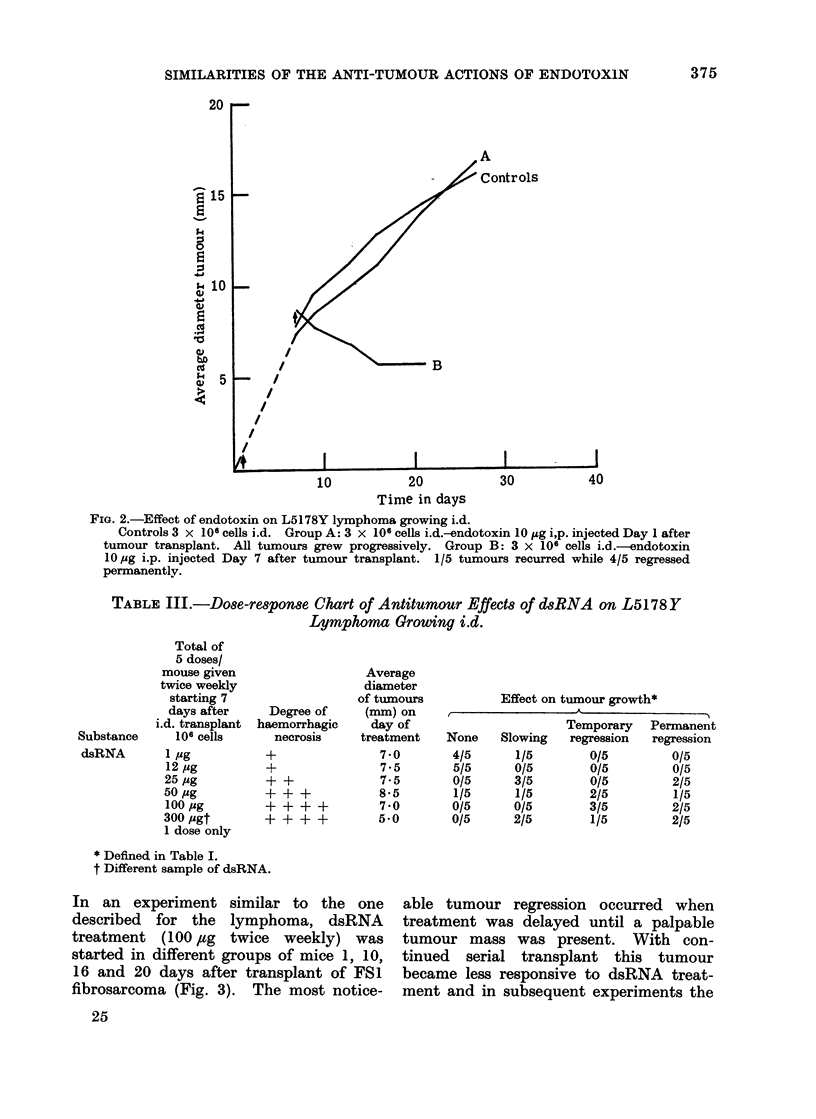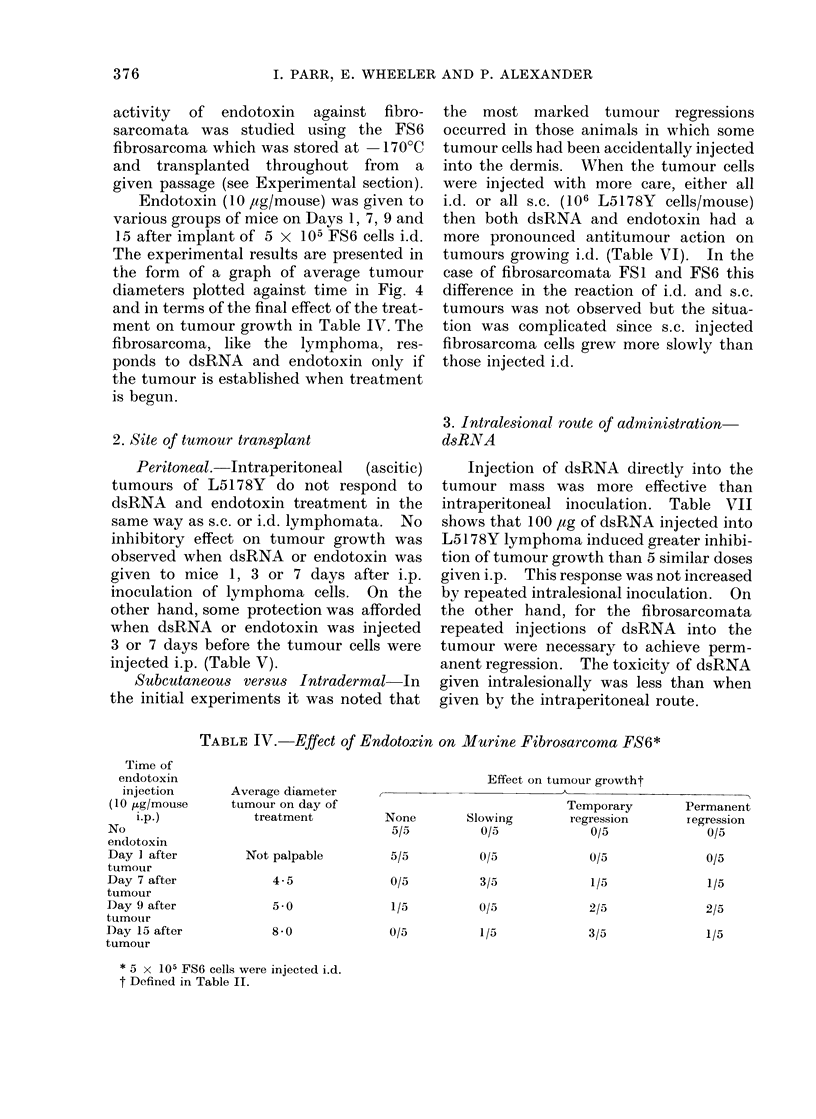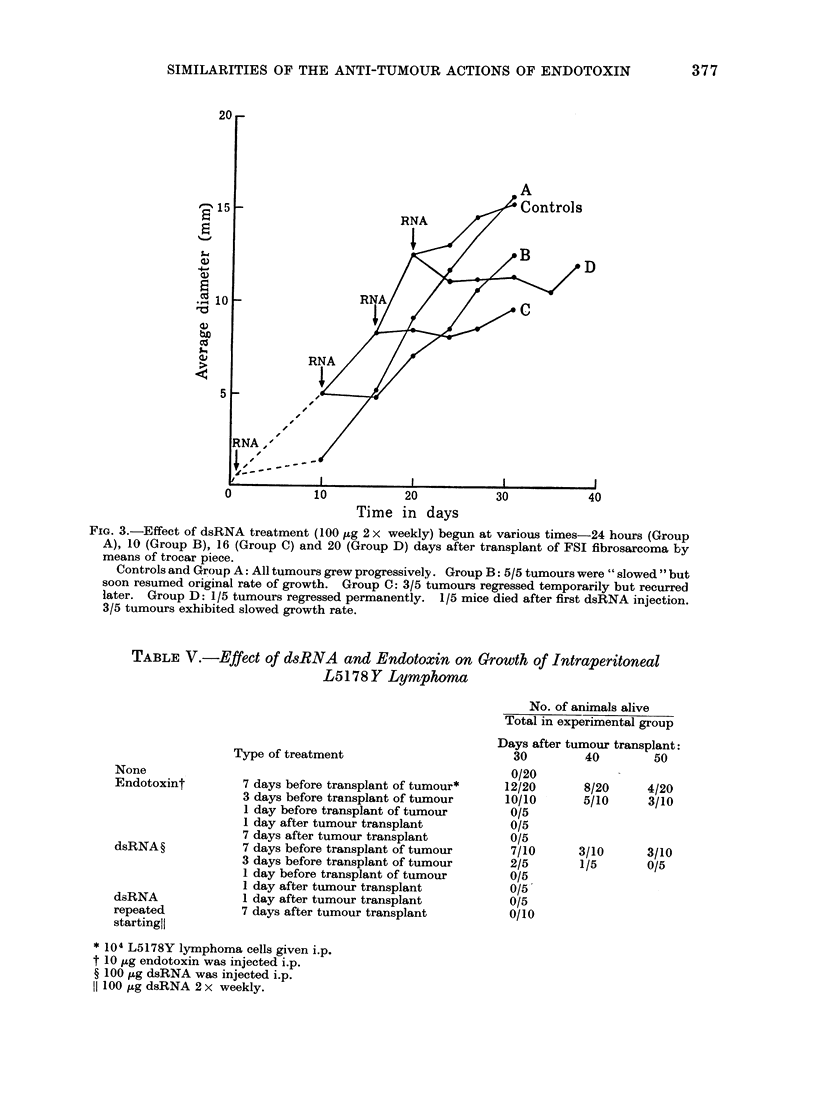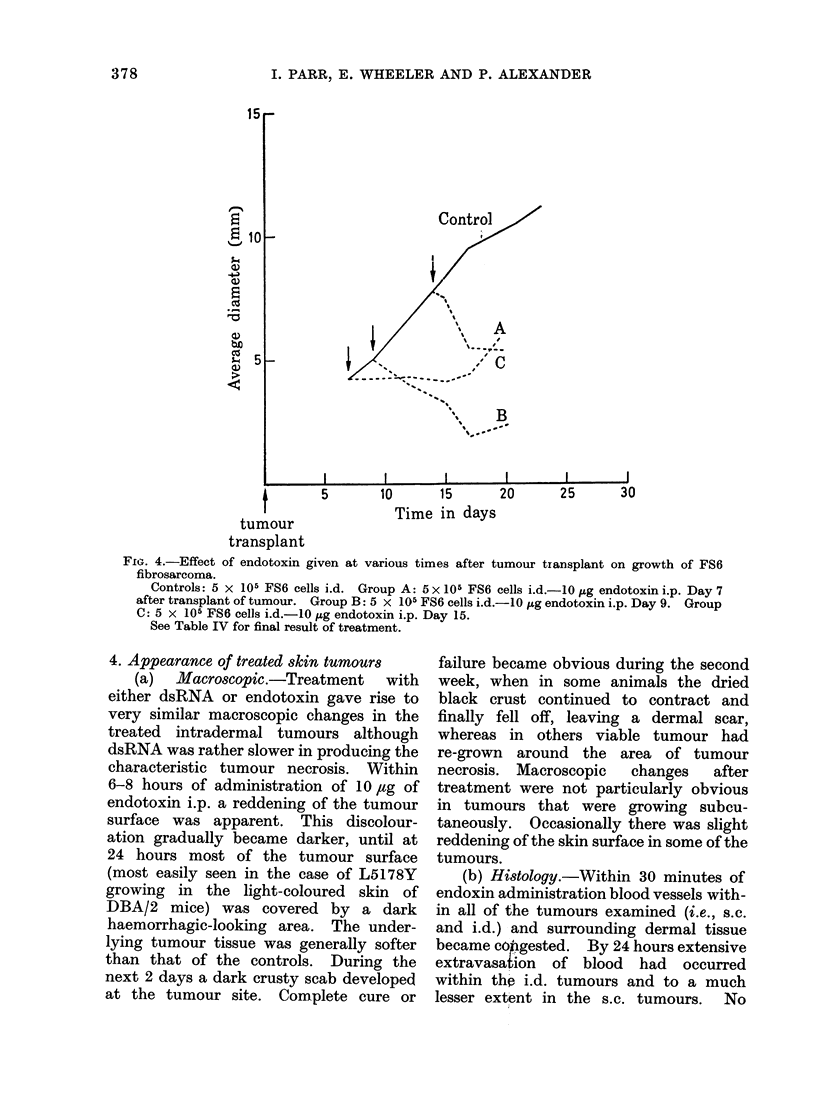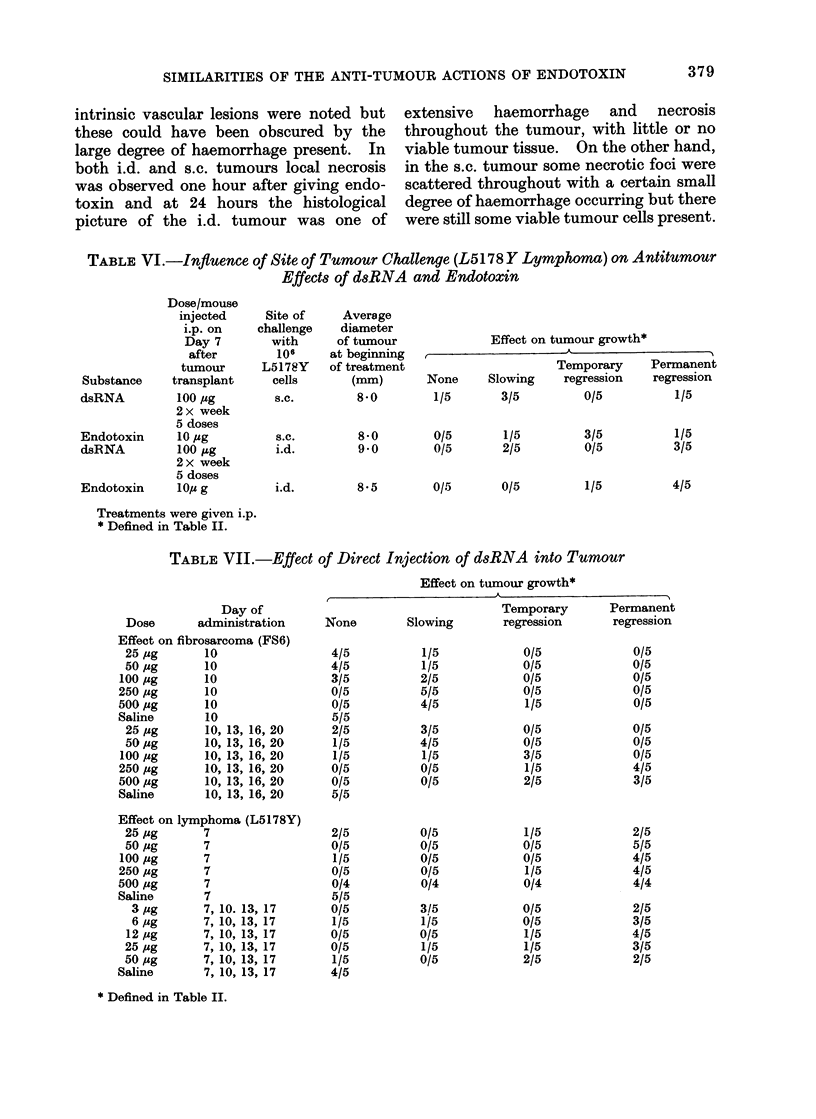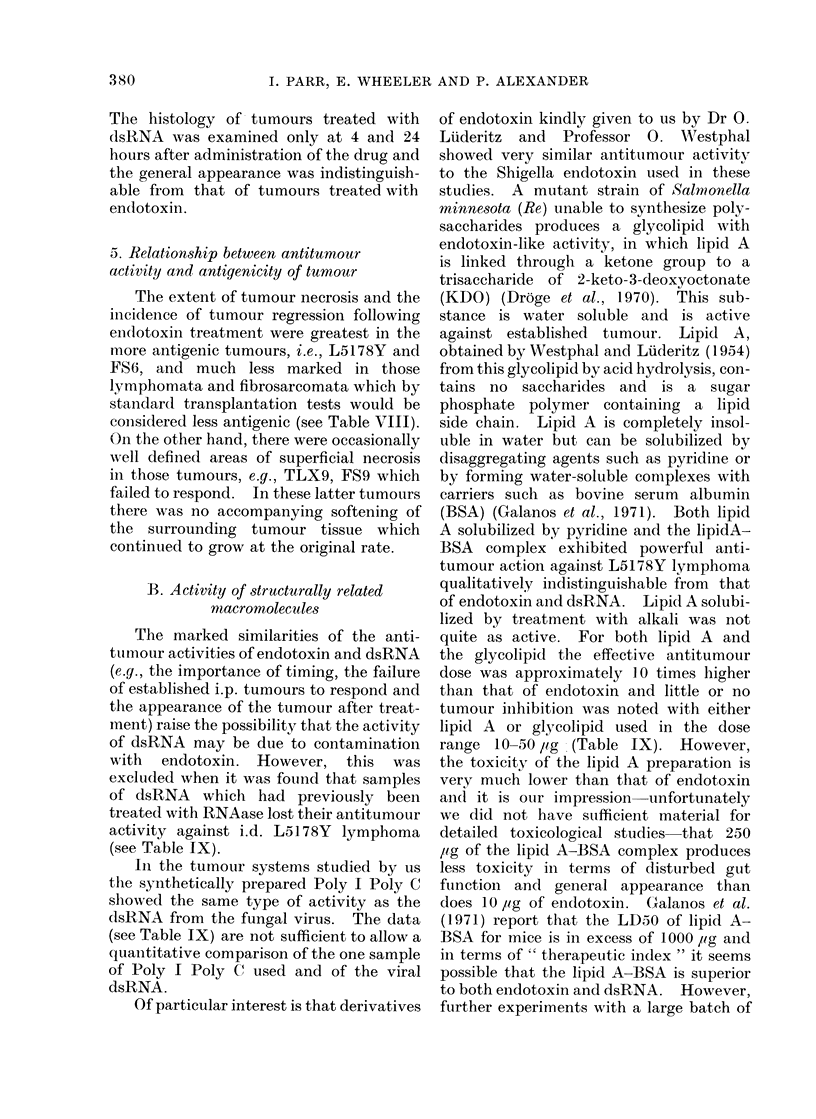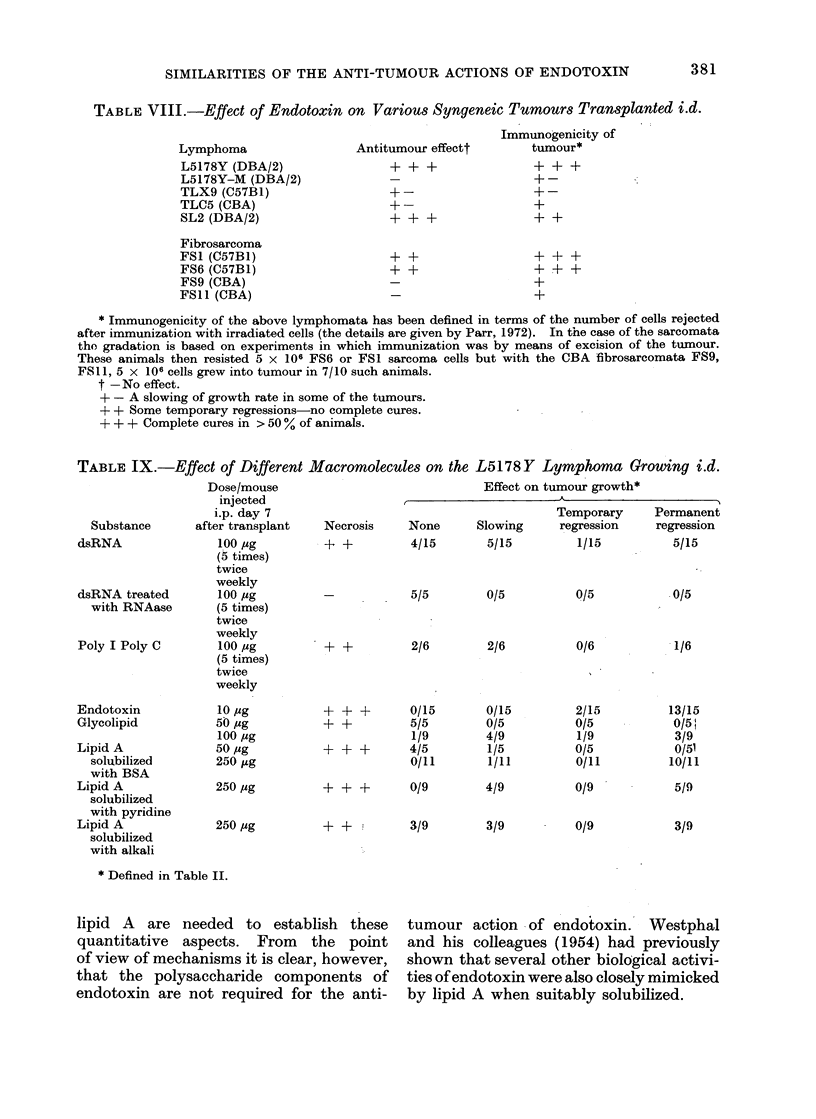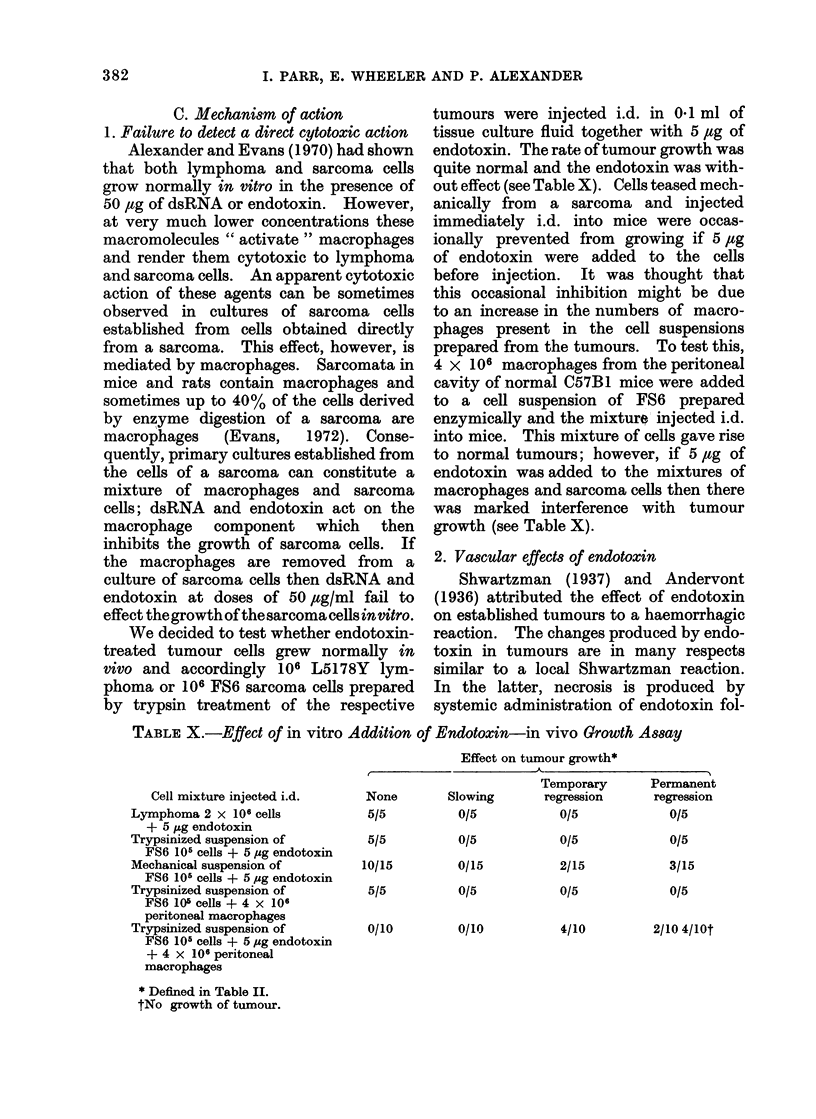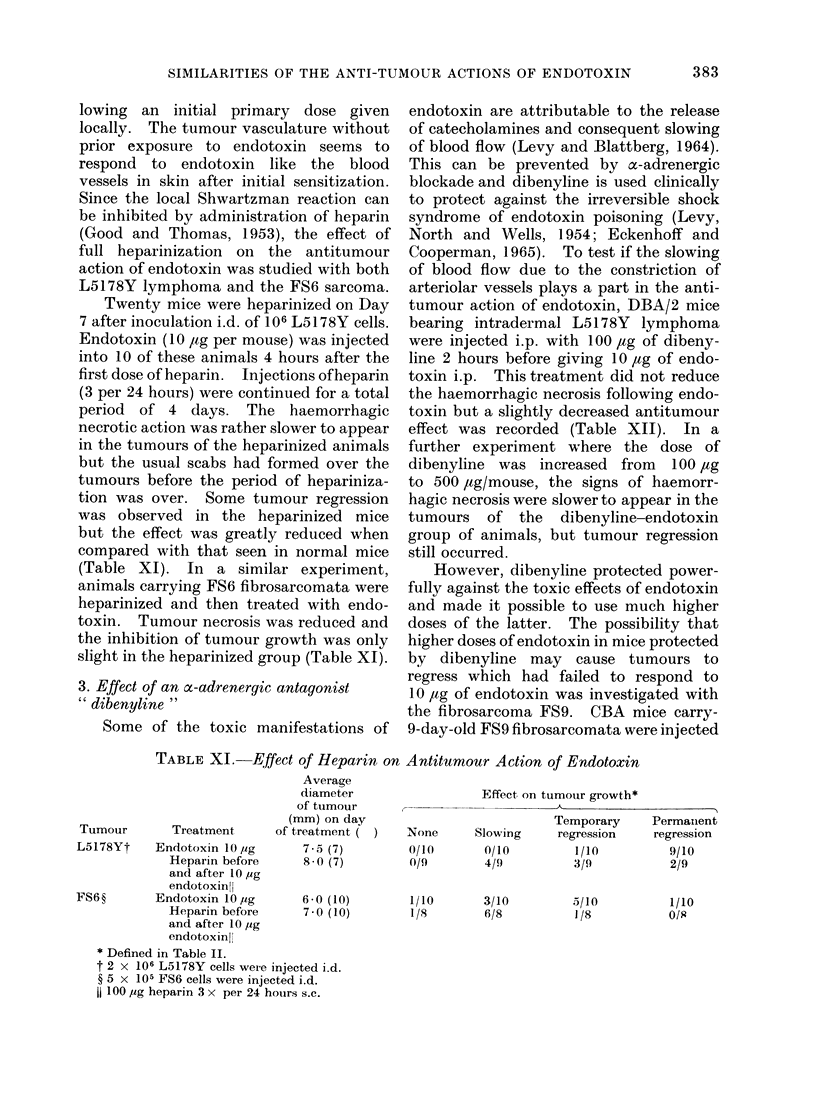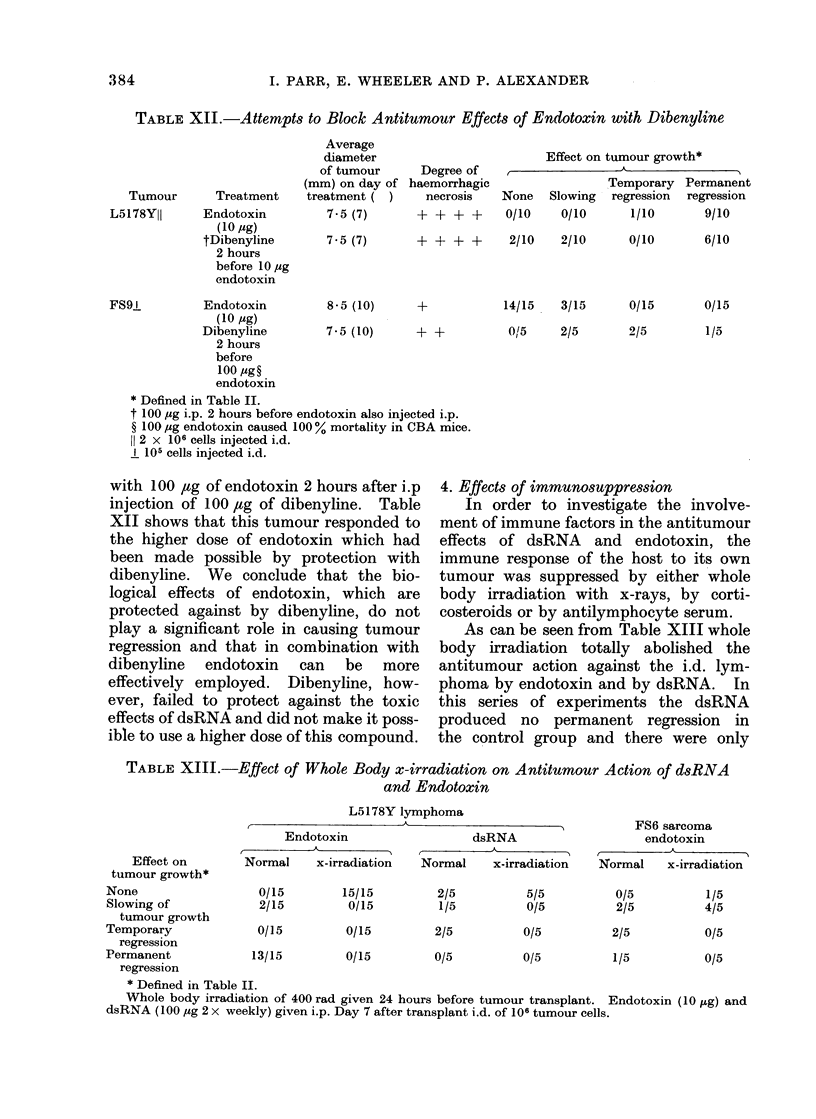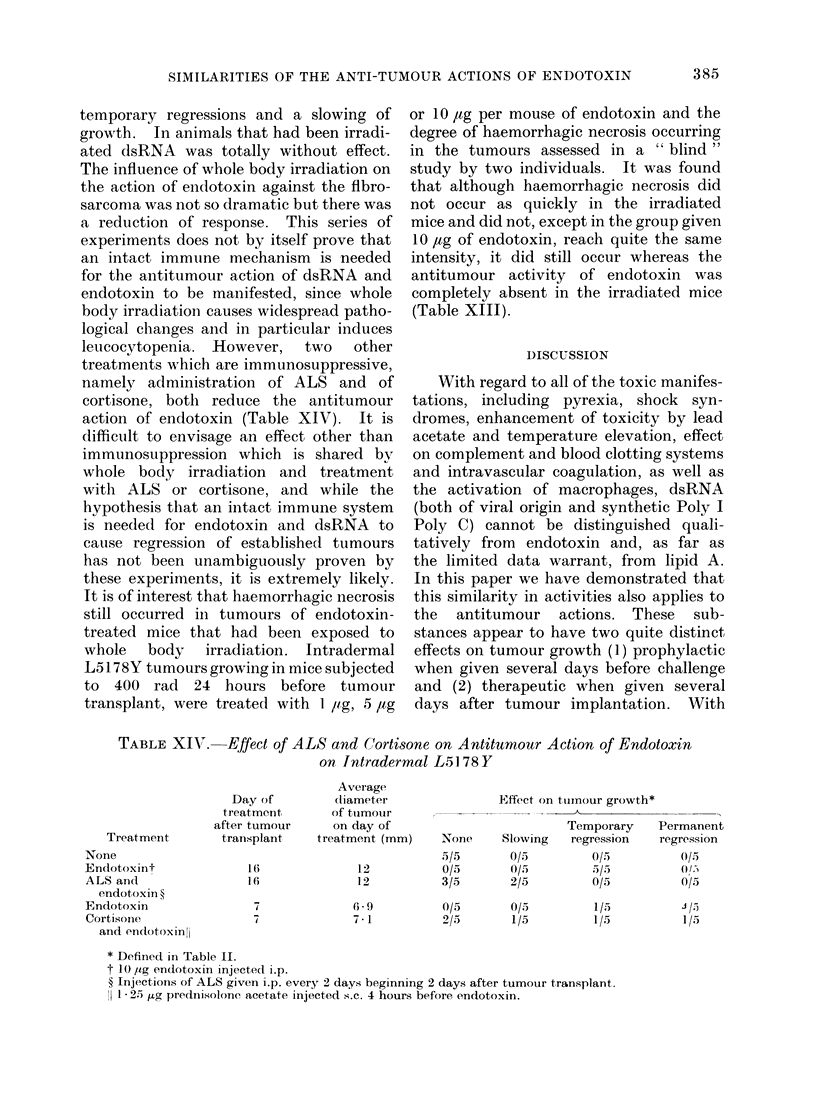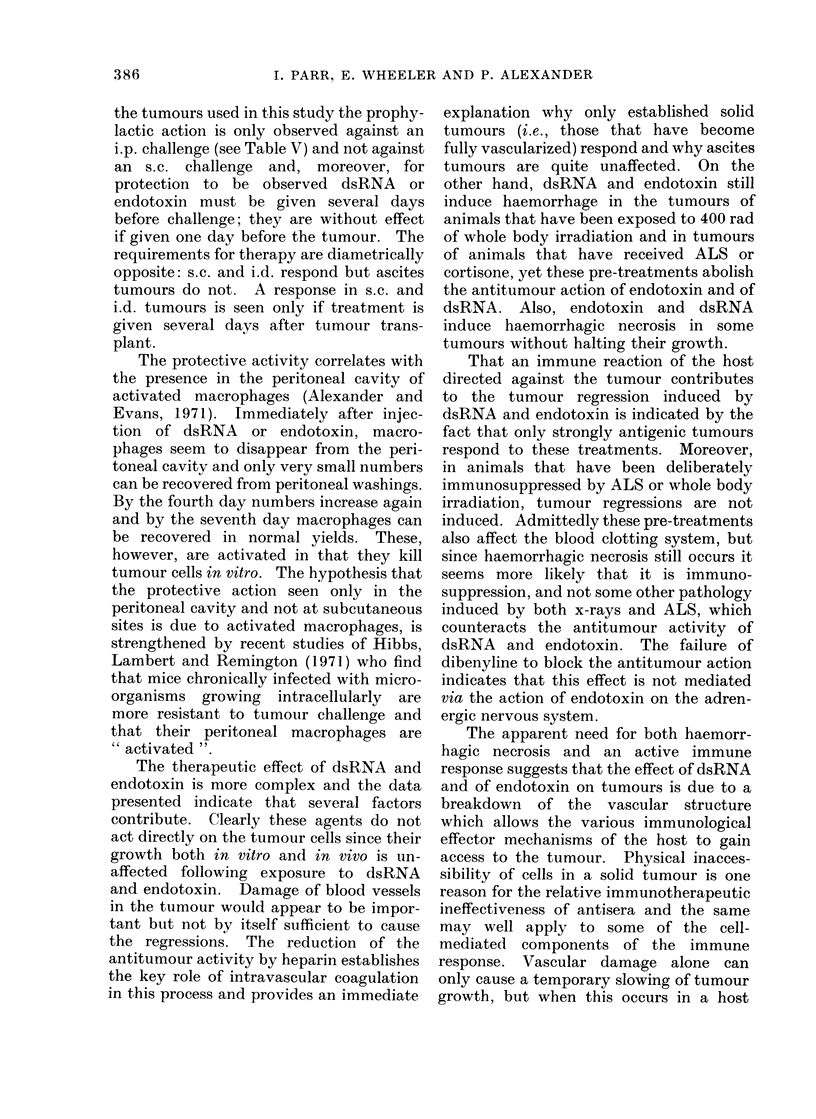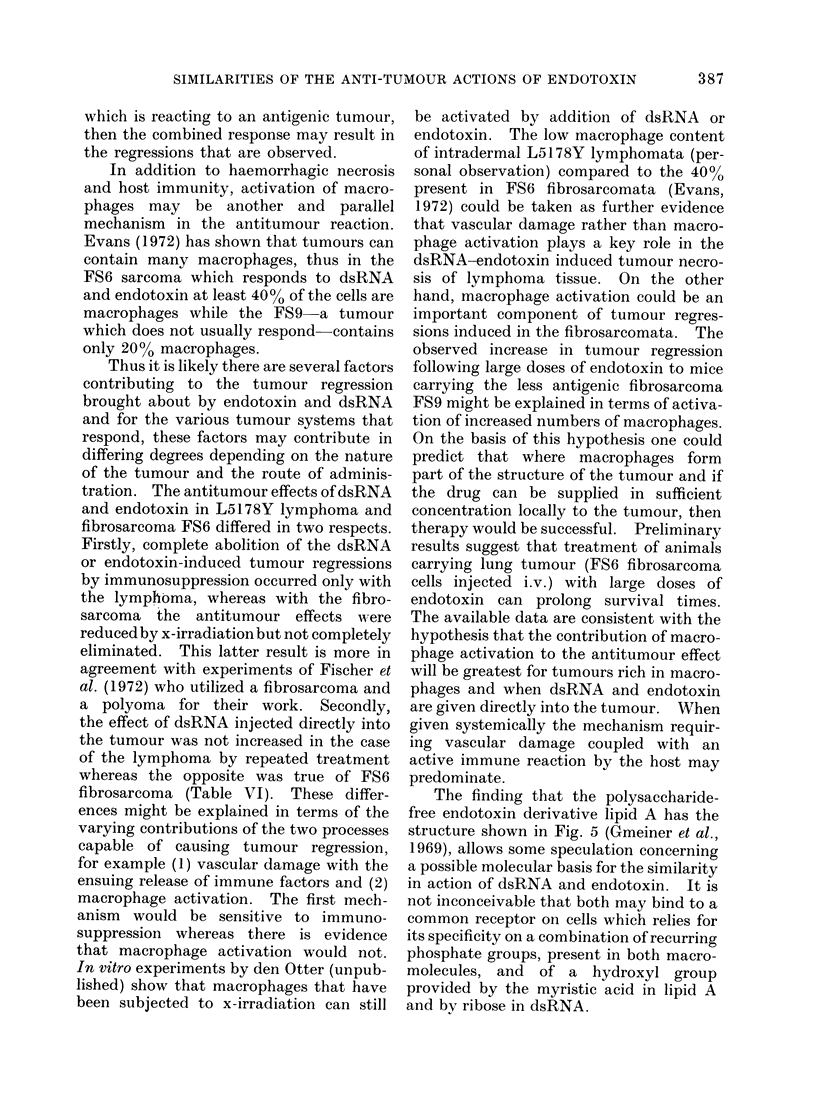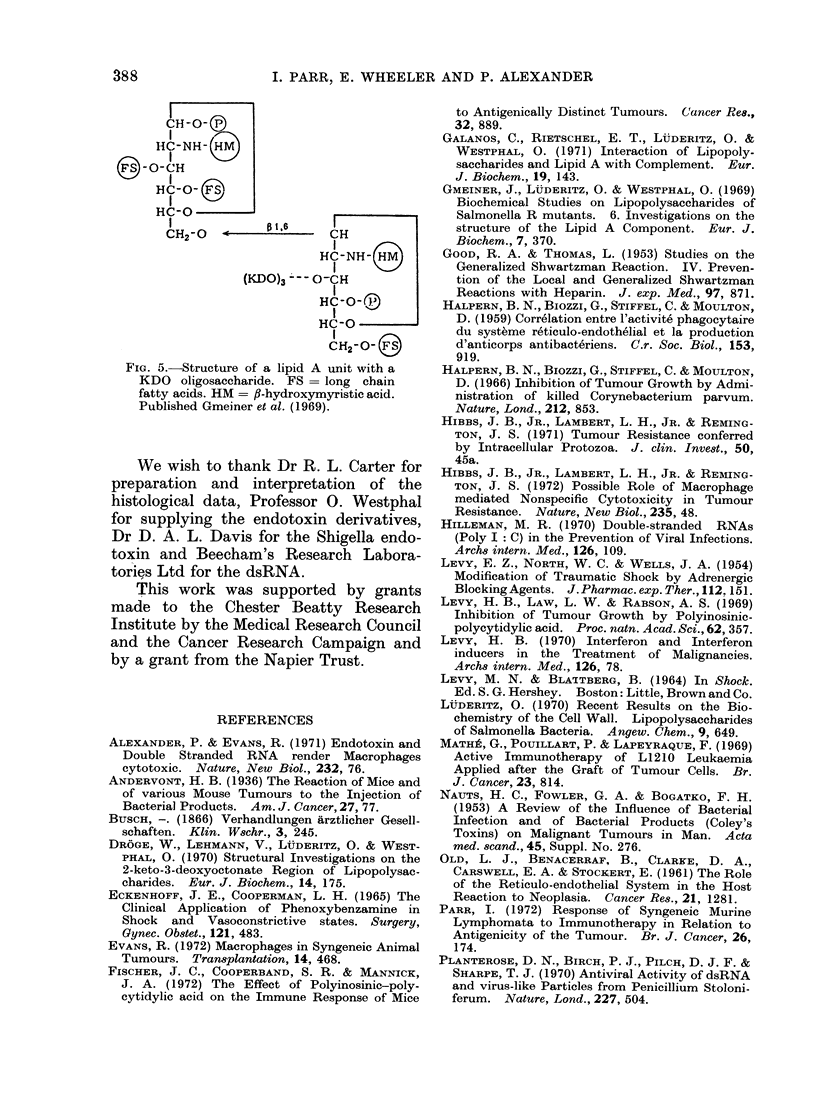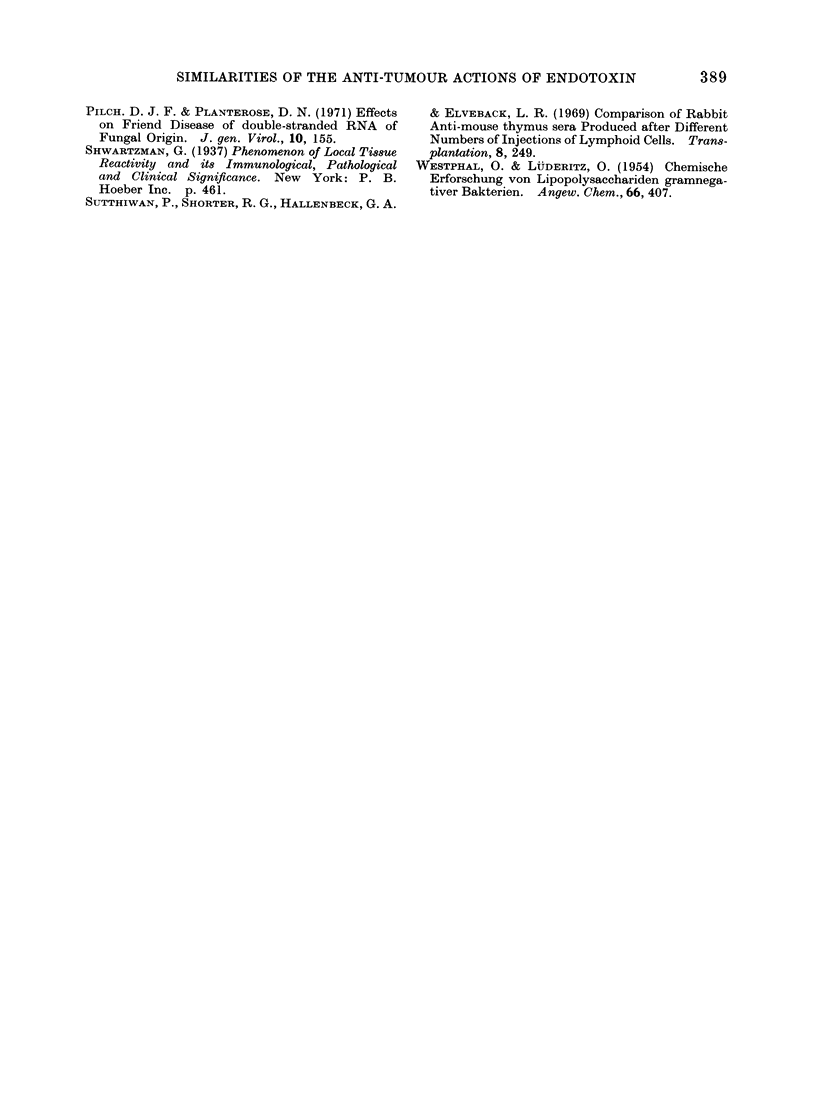# Similarities of the Anti-tumour Actions of Endotoxin, Lipid A and Double-stranded RNA

**DOI:** 10.1038/bjc.1973.45

**Published:** 1973-05

**Authors:** I. Parr, E. Wheeler, P. Alexander

## Abstract

Double stranded RNA (dsRNA) whether isolated from a fungal virus or prepared synthetically (*i.e.,* Poly I Poly C) and endotoxin were found to exert very similar effects on syngeneic murine lymphomata and fibrosarcomata. They cause complete regressions of some established subcutaneous (s.c.) or intradermal (i.d.) tumours but not of intraperitoneal (i.p.) tumours when administered either systemically or directly into the tumour. To achieve this effect the tumours must be fully established and the best results were obtained when treatment was started 7 days after transplant. If treatment is started within the first 3 days following the transplantation of the tumour then only a slight inhibition of growth rate was observed. These agents can also act prophylactically and protect mice against a subsequent challenge but only if this is given i.p. and not if given s.c. or i.d. The prophylactic action is explained by the action of dsRNA and endotoxin on peritoneal macrophages which cause them to become cytotoxic to tumour cells (*i.e.,* to become activated).

The therapeutic effect of systemically administered endotoxin and dsRNA on established tumours is not the result of a direct action on the tumour cells themselves but is a complex process requiring the co-operation of several host factors. Haemorrhagic necrosis involving coagulation is essential (*i.e.,* heparinization reduces the regression of tumours) but is not itself sufficient. Immunosuppression by whole body irradiation or by antilymphocyte serum also interferes with the antitumour action of dsRNA and endotoxin in spite of the fact that haemorrhagic necrosis still occurs. Also, the magnitude of the antitumour action correlated in a series of different tumours with their antigenicity. The observed tumour regressions are probably brought about by (1) vascular damage in the tumour which permits immune defence mechanisms of the host to gain access to the tumour and (2) activation of macrophages present within the tumour. The relative contribution of these two mechanisms may depend on the nature of the tumour and the route of administration of the active agents.

Dibenyline, which protects against the lethal action of endotoxin by preventing the action of the catecholamines on the α-adrenergic receptors, makes it possible to increase the effectiveness of endotoxin in tumours by allowing a large dose to be given. Lipid A, a derivative of endotoxin which does not contain polysaccharide, has similar antitumour action to dsRNA and endotoxin. Some common features of the chemical structure of lipid A and dsRNA are discussed.


*Br. J. Cancer* (1973) **27**, 370

### SIMILARITIES OF THE ANTI-TUMOUR ACTIONS OF ENDOTOXIN, LIPID A AND DOUBLE-STRANDED RNA

I. PARR, E. WHEELER AND P. ALEXANDER

*From the Chester Beatty Research Institute, Clifton Avenue, Belmont, Sutton, Surrey*

Received 31 January 1973. Accepted 19 February 1973

**Summary.—Double stranded RNA (dsRNA) whether isolated from a fungal virus or prepared synthetically (*i.e.*, Poly I Poly C) and endotoxin were found to exert very similar effects on syngeneic murine lymphomata and fibrosarcomata. They cause complete regressions of some established subcutaneous (s.c.) or intradermal (i.d.) tumours but not of intraperitoneal (i.p.) tumours when administered either systemically or directly into the tumour. To achieve this effect the tumours must be fully established and the best results were obtained when treatment was started 7 days after transplant. If treatment is started within the first 3 days following the transplantation of the tumour then only a slight inhibition of growth rate was observed. These agents can also act prophylactically and protect mice against a subsequent challenge but only if this is given i.p. and not if given s.c. or i.d. The prophylactic action is explained by the action of dsRNA and endotoxin on peritoneal macrophages which cause them to become cytotoxic to tumour cells (*i.e.*, to become activated).**

**The therapeutic effect of systemically administered endotoxin and dsRNA on established tumours is not the result of a direct action on the tumour cells themselves but is a complex process requiring the co-operation of several host factors. Haemorrhagic necrosis involving coagulation is essential (*i.e.*, heparinization reduces the regression of tumours) but is not itself sufficient. Immunosuppression by whole body irradiation or by antilymphocyte serum also interferes with the antitumour action of dsRNA and endotoxin in spite of the fact that haemorrhagic necrosis still occurs. Also, the magnitude of the antitumour action correlated in a series of different tumours with their antigenicity. The observed tumour regressions are probably brought about by (1) vascular damage in the tumour which permits immune defence mechanisms of the host to gain access to the tumour and (2) activation of macrophages present within the tumour. The relative contribution of these two mechanisms may depend on the nature of the tumour and the route of administration of the active agents.**

**Dibenyline, which protects against the lethal action of endotoxin by preventing the action of the catecholamines on the α-adrenergic receptors, makes it possible to increase the effectiveness of endotoxin in tumours by allowing a large dose to be given. Lipid A, a derivative of endotoxin which does not contain polysaccharide, has similar antitumour action to dsRNA and endotoxin. Some common features of the chemical structure of lipid A and dsRNA are discussed.**

Over a hundred years ago (Busch 1866), a Viennese physician noted that regressions of some malignant tumours appeared to coincide with the occurrence of erysipelas. Coley, having made a similar observation, deliberately treated patients with bacterial culture fluids (Coley's toxin), which contained endotoxins amongst many other components, and achieved some very striking results (*cf.* review by Nauts, Fowler and Bogatko, 1953). The subject of the antitumour action of endotoxins has attracted much attention during the whole of this century, but from an experimental standpoint, the key publication is that of Andervont (1936), in which he showed that large primary chemically-induced epitheliomata of mice regressed completely following systemic treatment with endotoxin and that these striking effects were associated with a haemorrhagic reaction and resultant damage of the tumour vasculature. Using primary as well as transplanted tumours in syngeneic systems, Andervont found that endotoxin was effective only after tumours had become fully established and was inactive when given immediately after a transplant. Double-stranded RNA (dsRNA) both of natural origin (*e.g.*, when isolated from fungal viruses) and when prepared synthetically (*i.e.*, Poly I Poly C) mimics many of the biological actions of endotoxin, such as toxicity and interferon induction. Both types of dsRNA have also been found to slow the growth of a variety of transplanted mouse tumours and leukaemias (Levy, Law and Rabson, 1969; Pilch and Planterose, 1971). Typical endotoxin-type antitumour action (*i.e.*, an effect which predominantly affected established tumour) had been recognized for dsRNA by Pilch and Planterose (1971) but in most of the other studies this was not noted because in general the dsRNA was administered soon after transplantation of the tumours.

Our interest in the antitumour activities of endotoxin and of dsRNA derived from the observation that after exposure to either of these substances *in vivo* or *in vitro*, peritoneal macrophages acquired the capacity to inhibit the growth of lymphoma and sarcoma cells (Alexander and Evans, 1971). Neither endotoxin nor dsRNA, at the concentrations used to treat macrophages, have a direct effect on the growth of tumour cells *in vitro*. The term "activated" has been used to describe macrophages that have acquired the capacity to inhibit *in vitro* the growth of tumour cells that are in direct contact with them and they need to be distinguished from "armed" macrophages which kill tumour cells in an immunologically specific way. Since the growth inhibitory effect of activated macrophages is confined to tumour cells (Hibbs, Lambert and Remmington, 1972), it seemed possible that such macrophages might be concerned with the *in vivo* antitumour activity of dsRNA and endotoxin.

The aim of the present study was to determine the conditions under which dsRNA of fungal origin and endotoxin inhibit the growth of established tumours. The induction of interferon by dsRNA may account for its inhibition of viral carcinogenesis (*cf.* review by Hilleman, 1970), but cannot explain its action on growing tumours *in vivo*. Both dsRNA and endotoxin have been shown under some conditions to act as immune adjuvants and to cause a nonspecific stimulation of the immune systems, and Levy (1970) has attributed the effect of dsRNA as an antitumour agent to this property. While it has been clearly shown that much more potent stimulants of the immune system than dsRNA, such as mycobacteria (*e.g.*, BCG) and *Corynebacterium parvum*, render rodents more resistant to a subsequent challenge with tumour cells (Halpern *et al.*, 1959, 1966; Old *et al.*, 1961), such agents have only a minor therapeutic effect when given after the tumour has been injected (Mathé, Pouillant and Rapeyraque, 1969; Parr, 1972). They are without effect on established tumours, and are active only in an animal with pre-existing tumour when the number of such tumour cells is very small. On the other hand, endotoxin, as first shown by Andervont (1936), and dsRNA, as shown in this paper, are most effective against established tumours and almost ineffective when there are only a few tumour cells present in the animal.

It would seem that mechanisms other than induction of interferon or non-specific stimulation of the immune system must come into play in the pronounced antitumour action shown by dsRNA and endotoxin against solid tumours.

Structurally, these two classes of macromolecules are very different. However, Lüderitz and colleagues (*cf.* review 1970) have shown convincingly that a polysaccharide-free derivative of endotoxin referred to as lipid A will, if suitably solubilized, mimic many of the biological actions of endotoxin. This applies also to the activation of macrophages (Alexander and Evans, 1971) and, as shown in the present investigation, to the anti-tumour action *in vivo*. Possible structural similarities of dsRNA and Lipid A are referred to in the Discussion.

### MATERIALS AND METHODS

*Mice.*—Ten-week old mice, CBA, C57B1 and DBA/2, were supplied from breeding colonies maintained in the Institute under specific pathogen-free conditions.

*Tumours.*—Two tumours, a lymphoma L5178Y and a fibrosarcoma FS6, were used for the majority of the experiments. Brief details of these and the other tumour lines used are given in Table I. The lymphomata were maintained in the ascitic form by serial weekly passage of washed, counted tumour cells into the strain of origin. Transplant of the fibrosarcomata was fortnightly by means of trocar fragments. At 4–5 monthly intervals the tumours were re-established from stock tumour cell lines maintained in liquid nitrogen. Single cell suspensions were prepared from the fibrosarcomata by enzymic digestion of minced tissues with TC.199 medium containing 0·13% trypsin, 0·13% collagenase and 0·002% DNAase for 15 min at room temperature.

Subcutaneous or intradermal challenges were made into the right flank of the animal. Growth of tumour was followed by bi-weekly measurement of two diameters by means of calipers.

dsRNA, which was kindly supplied by Beecham's Research Laboratories, was from a fungal virus obtained from a penicillin mould (Planterose *et al.*, 1970). Poly I Poly C was obtained from Miles Laboratories Inc. (Enhard, Indiana). Endotoxin was a highly purified water-soluble preparation of lipopolysaccharide from *Shigella* which was kindly donated to us by Dr D. A. L. Davies.

All the above 3 substances were injected in saline or TC.199 in volumes of 0·1 ml and, except where stated, were given i.p.

The following endotoxin derivatives were kindly donated by Professor O. Westphal (of the Max Planck Institute for Immunobiology, Freiburg, Germany) and are described by Galanos *et al.* (1971): A glycolipid from *Salmonella minnesota* (R595) which was dissolved in water by heating in a water bath for 2 minutes. Lipid A from *S. minnesota* (R345) which had been rendered soluble in water by 3 methods: with the aid of pyridine, by treatment with NaOH (1 hour at 56°C) and by complexing with bovine serum albumin (BSA) in a 1·2 : 1 BSA/Lipid A ratio. Isotonic solutions for injection (i.p.) were prepared by addition of concentrated saline to solutions of Lipid A in water. Dibenyline was obtained from Smith, Kline and French laboratories. Mice received 100 $\mu$g/animal in volumes of 1 ml injected i.p. 2 hours before administration of endotoxin. Mice received 3 injections (s.c.) each of 100 iu heparin per 24 hours. Fine needles (gauge 30) were used to minimize the numbers of deaths from bleeding.

Antilymphocyte serum was prepared by sensitizing rabbits with mouse thymocytes according to the method described by Sutthiwan *et al.* (1969). Eight injections of 0·1 ml of ALS were given i.p. on alternate days, beginning 2 days after tumour transplant.

TABLE I.—*Summary of Details of Origin of Tumours Described in Text*

| Tumour | Type | Mouse of origin | Method of induction | Date |
|---|---|---|---|---|
| L5178Y | Lymphoma | DBA/2 | Methylcholanthrene | 1961 |
| L5178Y-M | Lymphoma | DBA/2 | Arose from L5178Y | 1968 |
| SL2 | Lymphoma | DBA/2 | Spontaneous | 1968 |
| TCL5 | Lymphoma | CBA | Methylcholanthrene | 1968 |
| TLX9 | Lymphoma | C57B1/6 | Whole body x-irradiation | 1967 |
| FS1 | Fibrosarcoma | C57B1/6 | Benzpyrene pellet | 1969 |
| FS6 | Fibrosarcoma | C57B1/6 | Benzpyrene pellet | 1970 |
| FS9 | Fibrosarcoma | CBA | Benzpyrene pellet | 1970 |
| FS11 | Fibrosarcoma | CBA | Benzpyrene pellet | 1970 |

Whole body x-irradiation of 400 rad at the rate of 83 rad/min was delivered from a 220 kVp Marconi unit without filtration.

Prednisolone acetate was obtained from Roussel Laboratories, England. Mice were injected s.c. with 1·2 mg of prednisolone acetate 24 hours before receiving endotoxin.

### RESULTS

In the first series of experiments tumour cells were injected under the skin of the mouse and tumour growth was partly i.d. and partly s.c. In later experiments (see page 376) care was taken to inject the cells either wholly below (*i.e.*, s.c.) or wholly above (*i.e.*, i.d.) the muscle layer—*panniculus carnosus*—present in the skin of all rodents.

### A. *Effect of dsRNA and endotoxin on established s.c.–i.d. lymphomata and sarcomata*

#### 1. *Timing of dsRNA and endotoxin*

*Lymphoma.*—Neither dsRNA nor endotoxin (injected i.p. or i.v.) given before inoculation of tumour cells s.c. (in the flank) had any effect on the growth of the L5178Y lymphoma in syngeneic mice (Table II). A significant effect on the growth of a s.c. tumour was observed only when the treatment with dsRNA or endotoxin was delayed until a palpable tumour mass was present. This is illustrated in Fig. 1 in which one group of mice were injected i.p. with 100 $\mu$g of dsRNA twice weekly beginning 24 hours after transplant of $10^6$ L5178Y cells s.c., while in another group of animals treatment was delayed until 7 days after tumour transplant, by which time the tumours were 8 mm average diameter. In the first group there was little inhibition of tumour growth, whereas in the second, tumour necrosis occurred within 24 hours of treatment followed by tumour regression. In Fig. 1 the use of " average diameters " to record the response of the group treated on Day 7 became meaningless after 25 days because mice divided into two distinct categories—animals in which tumours had completely regressed and mice with re-growing tumours. No tumour necrosis or regression was noted in the group of mice treated from Day 1 onwards in spite of the dsRNA injections being carried on over the same period as the group started Day 7. Endotoxin caused tumour regression of a solid tumour only if administered after the tumour had become established and presumably vascularized. Fig. 2 illustrates an experiment in which a single dose of endotoxin was given either one or 7 days after the inoculation of tumour cells.

The dose response curve for the activity of dsRNA against an i.d. L5178Y lymphoma plateaus between doses of 50 $\mu$g and 100 $\mu$g given twice weekly (Table III). Five doses of 100 $\mu$g of dsRNA given twice weekly were close to the maximum tolerated dose and 150 $\mu$g caused some deaths. While a single injection of 100 $\mu$g of dsRNA had no detectable effect on tumour growth, one of 300 $\mu$g caused tumour regression (Table III). This dose, however, could not be tolerated under all conditions since environmental factors affect toxicity. Both the toxicity and antitumour activity of endotoxin are very dependent on the temperature at which the animals are maintained and no attempt was made to establish a dose-response curve. Under normal conditions in the animal house (*i.e.*, 25°C) 10 $\mu$g of endotoxin regularly caused tumour regression. Increasing the dose to 15, 20 and 25 $\mu$g did not improve the antitumour effect and the latter made the mice obviously sick. Consequently a dose of 10 $\mu$g/mouse was used throughout. However, under conditions of above average environmental temperature (*i.e.*, in the range 26–29°C) 1 $\mu$g endotoxin per mouse was sufficient to induce permanent tumour regression in 4/5 animals. Attempts to give repeated doses of 10 $\mu$g of endotoxin resulted in the death of 50% of the animals.

*Fibrosarcomata.*—The initial experiments were made on mice carrying trocar fragments of tumour placed in the flank.

FIG. 1.—Effect of dsRNA on L5178Y growing in skin as solid lymphoma. $10^6$ L5178Y cells injected s.c.–i.d. dsRNA 100 $\mu$g 2 × weekly injected i.p. beginning (A) 24 hours after tumour transplant and (B) 7 days after transplant.

Controls and Group A: All tumours grew progressively into large masses. Group B: 3/5 of the tumours recurred and grew into large masses while 2/5 were in total regression on Day 40 when the experiment was terminated.

TABLE II.—*Effect of dsRNA and Endotoxin Given at Various Times Before and After Implant of L5178Y s.c.–i.d. in Skin of Syngeneic (DBA/2) Mice*

| Time of administration | Treatment | Effect on tumour growth: None | Slowing* | Temporary regression† | Permanent regression§ |
|---|---|---|---|---|---|
| 7 days before | Endotoxin | 5/5 | 0/5 | 0/5 | 0/5 |
| implant | dsRNA | 5/5 | 0/5 | 0/5 | 0/5 |
| 3 days before | Endotoxin | 5/5 | 0/5 | 0/5 | 0/5 |
| implant | dsRNA | 5/5 | 0/5 | 0/5 | 0/5 |
| 1 day before | Endotoxin | 5/5 | 0/5 | 0/5 | 0/5 |
| implant | dsRNA | 5/5 | 0/5 | 0/5 | 0/5 |
| 1 day after | Endotoxin | 5/5 | 0/5 | 0/5 | 0/5 |
| implant | dsRNA | 5/5 | 0/5 | 0/5 | 0/5 |
| 7 days after | Endotoxin | 0/5 | 0/5 | 1/5 | 4/5 |
| implant | dsRNA | 0/5 | 0/5 | 3/5 | 2/5 |
| | None | 5/5 | | | |

$10^6$ L5178Y were injected s.c.–i.d. in the flank.
10 $\mu$g endotoxin given i.p.
100 $\mu$g dsRNA given i.p. bi-weekly for $2\frac{1}{2}$ weeks.
Figures denote $\frac{\text{No. of mice showing effect}}{\text{Total number in experiment}}$

* Slowing of growth was defined as tumour size not increasing for at least 5 days after treatment or decreases in average diameter < 2 mm.

† Temporary regression was defined as decrease in average diameter of tumour > 2 mm.

§ Permanent regression, survivors remained fit for 3 months after treatment.

FIG. 2.—Effect of endotoxin on L5178Y lymphoma growing i.d.
Controls 3 × $10^6$ cells i.d. Group A: 3 × $10^6$ cells i.d.-endotoxin 10 μg i,p. injected Day 1 after tumour transplant. All tumours grew progressively. Group B: 3 × $10^6$ cells i.d.—endotoxin 10 μg i.p. injected Day 7 after tumour transplant. 1/5 tumours recurred while 4/5 regressed permanently.

TABLE III.—*Dose-response Chart of Antitumour Effects of dsRNA on L5178Y Lymphoma Growing i.d.*

| Substance | Total of 5 doses/mouse given twice weekly starting 7 days after i.d. transplant $10^6$ cells | Degree of haemorrhagic necrosis | Average diameter of tumours (mm) on day of treatment | Effect on tumour growth* | | | |
|---|---|---|---|---|---|---|---|
| | | | | None | Slowing | Temporary regression | Permanent regression |
| dsRNA | 1 μg | + | 7·0 | 4/5 | 1/5 | 0/5 | 0/5 |
| | 12 μg | + | 7·5 | 5/5 | 0/5 | 0/5 | 0/5 |
| | 25 μg | + + | 7·5 | 0/5 | 3/5 | 0/5 | 2/5 |
| | 50 μg | + + + | 8·5 | 1/5 | 1/5 | 2/5 | 1/5 |
| | 100 μg | + + + + | 7·0 | 0/5 | 0/5 | 3/5 | 2/5 |
| | 300 μg† 1 dose only | + + + + | 5·0 | 0/5 | 2/5 | 1/5 | 2/5 |

* Defined in Table I.

† Different sample of dsRNA.

In an experiment similar to the one described for the lymphoma, dsRNA treatment (100 μg twice weekly) was started in different groups of mice 1, 10, 16 and 20 days after transplant of FS1 fibrosarcoma (Fig. 3). The most noticeable tumour regression occurred when treatment was delayed until a palpable tumour mass was present. With continued serial transplant this tumour became less responsive to dsRNA treatment and in subsequent experiments the activity of endotoxin against fibrosarcomata was studied using the FS6 fibrosarcoma which was stored at −170°C and transplanted throughout from a given passage (see Experimental section).

Endotoxin (10 $\mu$g/mouse) was given to various groups of mice on Days 1, 7, 9 and 15 after implant of $5 \times 10^5$ FS6 cells i.d. The experimental results are presented in the form of a graph of average tumour diameters plotted against time in Fig. 4 and in terms of the final effect of the treatment on tumour growth in Table IV. The fibrosarcoma, like the lymphoma, responds to dsRNA and endotoxin only if the tumour is established when treatment is begun.

### 2. *Site of tumour transplant*

*Peritoneal.*—Intraperitoneal (ascitic) tumours of L5178Y do not respond to dsRNA and endotoxin treatment in the same way as s.c. or i.d. lymphomata. No inhibitory effect on tumour growth was observed when dsRNA or endotoxin was given to mice 1, 3 or 7 days after i.p. inoculation of lymphoma cells. On the other hand, some protection was afforded when dsRNA or endotoxin was injected 3 or 7 days before the tumour cells were injected i.p. (Table V).

*Subcutaneous versus Intradermal*—In the initial experiments it was noted that the most marked tumour regressions occurred in those animals in which some tumour cells had been accidentally injected into the dermis. When the tumour cells were injected with more care, either all i.d. or all s.c. ($10^6$ L5178Y cells/mouse) then both dsRNA and endotoxin had a more pronounced antitumour action on tumours growing i.d. (Table VI). In the case of fibrosarcomata FS1 and FS6 this difference in the reaction of i.d. and s.c. tumours was not observed but the situation was complicated since s.c. injected fibrosarcoma cells grew more slowly than those injected i.d.

### 3. *Intralesional route of administration—dsRNA*

Injection of dsRNA directly into the tumour mass was more effective than intraperitoneal inoculation. Table VII shows that 100 $\mu$g of dsRNA injected into L5178Y lymphoma induced greater inhibition of tumour growth than 5 similar doses given i.p. This response was not increased by repeated intralesional inoculation. On the other hand, for the fibrosarcomata repeated injections of dsRNA into the tumour were necessary to achieve permanent regression. The toxicity of dsRNA given intralesionally was less than when given by the intraperitoneal route.

TABLE IV.—*Effect of Endotoxin on Murine Fibrosarcoma FS6**

| Time of endotoxin injection (10 $\mu$g/mouse i.p.) | Average diameter tumour on day of treatment | Effect on tumour growth† | | | |
|---|---|---|---|---|---|
| | | None | Slowing | Temporary regression | Permanent regression |
| No endotoxin | | 5/5 | 0/5 | 0/5 | 0/5 |
| Day 1 after tumour | Not palpable | 5/5 | 0/5 | 0/5 | 0/5 |
| Day 7 after tumour | 4·5 | 0/5 | 3/5 | 1/5 | 1/5 |
| Day 9 after tumour | 5·0 | 1/5 | 0/5 | 2/5 | 2/5 |
| Day 15 after tumour | 8·0 | 0/5 | 1/5 | 3/5 | 1/5 |

* $5 \times 10^5$ FS6 cells were injected i.d.

† Defined in Table II.

FIG. 3.—Effect of dsRNA treatment (100 μg 2 × weekly) begun at various times—24 hours (Group A), 10 (Group B), 16 (Group C) and 20 (Group D) days after transplant of FSI fibrosarcoma by means of trocar piece.

Controls and Group A: All tumours grew progressively. Group B: 5/5 tumours were "slowed" but soon resumed original rate of growth. Group C: 3/5 tumours regressed temporarily but recurred later. Group D: 1/5 tumours regressed permanently. 1/5 mice died after first dsRNA injection. 3/5 tumours exhibited slowed growth rate.

TABLE V.—*Effect of dsRNA and Endotoxin on Growth of Intraperitoneal L5178Y Lymphoma*

| | Type of treatment | No. of animals alive / Total in experimental group — Days after tumour transplant: 30 | 40 | 50 |
|---|---|---|---|---|
| None | | 0/20 | | |
| Endotoxin† | 7 days before transplant of tumour* | 12/20 | 8/20 | 4/20 |
| | 3 days before transplant of tumour | 10/10 | 5/10 | 3/10 |
| | 1 day before transplant of tumour | 0/5 | | |
| | 1 day after tumour transplant | 0/5 | | |
| | 7 days after tumour transplant | 0/5 | | |
| dsRNA§ | 7 days before transplant of tumour | 7/10 | 3/10 | 3/10 |
| | 3 days before transplant of tumour | 2/5 | 1/5 | 0/5 |
| | 1 day before transplant of tumour | 0/5 | | |
| | 1 day after tumour transplant | 0/5 | | |
| dsRNA repeated starting‖ | 1 day after tumour transplant | 0/5 | | |
| | 7 days after tumour transplant | 0/10 | | |

* $10^4$ L5178Y lymphoma cells given i.p.

† 10 μg endotoxin was injected i.p.

§ 100 μg dsRNA was injected i.p.

‖ 100 μg dsRNA 2 × weekly.

FIG. 4.—Effect of endotoxin given at various times after tumour transplant on growth of FS6 fibrosarcoma.

Controls: 5 × $10^5$ FS6 cells i.d. Group A: 5×$10^5$ FS6 cells i.d.—10 μg endotoxin i.p. Day 7 after transplant of tumour. Group B: 5 × $10^5$ FS6 cells i.d.—10 μg endotoxin i.p. Day 9. Group C: 5 × $10^5$ FS6 cells i.d.—10 μg endotoxin i.p. Day 15.

See Table IV for final result of treatment.

4. *Appearance of treated skin tumours*

(a) *Macroscopic.*—Treatment with either dsRNA or endotoxin gave rise to very similar macroscopic changes in the treated intradermal tumours although dsRNA was rather slower in producing the characteristic tumour necrosis. Within 6–8 hours of administration of 10 μg of endotoxin i.p. a reddening of the tumour surface was apparent. This discolouration gradually became darker, until at 24 hours most of the tumour surface (most easily seen in the case of L5178Y growing in the light-coloured skin of DBA/2 mice) was covered by a dark haemorrhagic-looking area. The underlying tumour tissue was generally softer than that of the controls. During the next 2 days a dark crusty scab developed at the tumour site. Complete cure or failure became obvious during the second week, when in some animals the dried black crust continued to contract and finally fell off, leaving a dermal scar, whereas in others viable tumour had re-grown around the area of tumour necrosis. Macroscopic changes after treatment were not particularly obvious in tumours that were growing subcutaneously. Occasionally there was slight reddening of the skin surface in some of the tumours.

(b) *Histology.*—Within 30 minutes of endoxin administration blood vessels within all of the tumours examined (*i.e.*, s.c. and i.d.) and surrounding dermal tissue became congested. By 24 hours extensive extravasation of blood had occurred within the i.d. tumours and to a much lesser extent in the s.c. tumours. No intrinsic vascular lesions were noted but these could have been obscured by the large degree of haemorrhage present. In both i.d. and s.c. tumours local necrosis was observed one hour after giving endotoxin and at 24 hours the histological picture of the i.d. tumour was one of extensive haemorrhage and necrosis throughout the tumour, with little or no viable tumour tissue. On the other hand, in the s.c. tumour some necrotic foci were scattered throughout with a certain small degree of haemorrhage occurring but there were still some viable tumour cells present.

TABLE VI.—*Influence of Site of Tumour Challenge (L5178Y Lymphoma) on Antitumour Effects of dsRNA and Endotoxin*

| Substance | Dose/mouse injected i.p. on Day 7 after tumour transplant | Site of challenge with $10^6$ L5178Y cells | Average diameter of tumour at beginning of treatment (mm) | Effect on tumour growth* | | | |
|---|---|---|---|---|---|---|---|
| | | | | None | Slowing | Temporary regression | Permanent regression |
| dsRNA | 100 μg 2× week 5 doses | s.c. | 8·0 | 1/5 | 3/5 | 0/5 | 1/5 |
| Endotoxin | 10 μg | s.c. | 8·0 | 0/5 | 1/5 | 3/5 | 1/5 |
| dsRNA | 100 μg 2× week 5 doses | i.d. | 9·0 | 0/5 | 2/5 | 0/5 | 3/5 |
| Endotoxin | 10μ g | i.d. | 8·5 | 0/5 | 0/5 | 1/5 | 4/5 |

Treatments were given i.p.
* Defined in Table II.

TABLE VII.—*Effect of Direct Injection of dsRNA into Tumour*

| Dose | Day of administration | Effect on tumour growth* | | | |
|---|---|---|---|---|---|
| | | None | Slowing | Temporary regression | Permanent regression |
| Effect on fibrosarcoma (FS6) | | | | | |
| 25 μg | 10 | 4/5 | 1/5 | 0/5 | 0/5 |
| 50 μg | 10 | 4/5 | 1/5 | 0/5 | 0/5 |
| 100 μg | 10 | 3/5 | 2/5 | 0/5 | 0/5 |
| 250 μg | 10 | 0/5 | 5/5 | 0/5 | 0/5 |
| 500 μg | 10 | 0/5 | 4/5 | 1/5 | 0/5 |
| Saline | 10 | 5/5 | | | |
| 25 μg | 10, 13, 16, 20 | 2/5 | 3/5 | 0/5 | 0/5 |
| 50 μg | 10, 13, 16, 20 | 1/5 | 4/5 | 0/5 | 0/5 |
| 100 μg | 10, 13, 16, 20 | 1/5 | 1/5 | 3/5 | 0/5 |
| 250 μg | 10, 13, 16, 20 | 0/5 | 0/5 | 1/5 | 4/5 |
| 500 μg | 10, 13, 16, 20 | 0/5 | 0/5 | 2/5 | 3/5 |
| Saline | 10, 13, 16, 20 | 5/5 | | | |
| Effect on lymphoma (L5178Y) | | | | | |
| 25 μg | 7 | 2/5 | 0/5 | 1/5 | 2/5 |
| 50 μg | 7 | 0/5 | 0/5 | 0/5 | 5/5 |
| 100 μg | 7 | 1/5 | 0/5 | 0/5 | 4/5 |
| 250 μg | 7 | 0/5 | 0/5 | 1/5 | 4/5 |
| 500 μg | 7 | 0/4 | 0/4 | 0/4 | 4/4 |
| Saline | 7 | 5/5 | | | |
| 3 μg | 7, 10. 13, 17 | 0/5 | 3/5 | 0/5 | 2/5 |
| 6 μg | 7, 10, 13, 17 | 1/5 | 1/5 | 0/5 | 3/5 |
| 12 μg | 7, 10, 13, 17 | 0/5 | 0/5 | 1/5 | 4/5 |
| 25 μg | 7, 10, 13, 17 | 0/5 | 1/5 | 1/5 | 3/5 |
| 50 μg | 7, 10, 13, 17 | 1/5 | 0/5 | 2/5 | 2/5 |
| Saline | 7, 10, 13, 17 | 4/5 | | | |

* Defined in Table II.

The histology of tumours treated with dsRNA was examined only at 4 and 24 hours after administration of the drug and the general appearance was indistinguishable from that of tumours treated with endotoxin.

### 5. *Relationship between antitumour activity and antigenicity of tumour*

The extent of tumour necrosis and the incidence of tumour regression following endotoxin treatment were greatest in the more antigenic tumours, *i.e.*, L5178Y and FS6, and much less marked in those lymphomata and fibrosarcomata which by standard transplantation tests would be considered less antigenic (see Table VIII). On the other hand, there were occasionally well defined areas of superficial necrosis in those tumours, *e.g.*, TLX9, FS9 which failed to respond. In these latter tumours there was no accompanying softening of the surrounding tumour tissue which continued to grow at the original rate.

### B. *Activity of structurally related macromolecules*

The marked similarities of the antitumour activities of endotoxin and dsRNA (*e.g.*, the importance of timing, the failure of established i.p. tumours to respond and the appearance of the tumour after treatment) raise the possibility that the activity of dsRNA may be due to contamination with endotoxin. However, this was excluded when it was found that samples of dsRNA which had previously been treated with RNAase lost their antitumour activity against i.d. L5178Y lymphoma (see Table IX).

In the tumour systems studied by us the synthetically prepared Poly I Poly C showed the same type of activity as the dsRNA from the fungal virus. The data (see Table IX) are not sufficient to allow a quantitative comparison of the one sample of Poly I Poly C used and of the viral dsRNA.

Of particular interest is that derivatives of endotoxin kindly given to us by Dr O. Lüderitz and Professor O. Westphal showed very similar antitumour activity to the Shigella endotoxin used in these studies. A mutant strain of *Salmonella minnesota* (*Re*) unable to synthesize polysaccharides produces a glycolipid with endotoxin-like activity, in which lipid A is linked through a ketone group to a trisaccharide of 2-keto-3-deoxyoctonate (KDO) (Dröge *et al.*, 1970). This substance is water soluble and is active against established tumour. Lipid A, obtained by Westphal and Lüderitz (1954) from this glycolipid by acid hydrolysis, contains no saccharides and is a sugar phosphate polymer containing a lipid side chain. Lipid A is completely insoluble in water but can be solubilized by disaggregating agents such as pyridine or by forming water-soluble complexes with carriers such as bovine serum albumin (BSA) (Galanos *et al.*, 1971). Both lipid A solubilized by pyridine and the lipidA–BSA complex exhibited powerful antitumour action against L5178Y lymphoma qualitatively indistinguishable from that of endotoxin and dsRNA. Lipid A solubilized by treatment with alkali was not quite as active. For both lipid A and the glycolipid the effective antitumour dose was approximately 10 times higher than that of endotoxin and little or no tumour inhibition was noted with either lipid A or glycolipid used in the dose range 10–50 $\mu$g (Table IX). However, the toxicity of the lipid A preparation is very much lower than that of endotoxin and it is our impression—unfortunately we did not have sufficient material for detailed toxicological studies—that 250 $\mu$g of the lipid A–BSA complex produces less toxicity in terms of disturbed gut function and general appearance than does 10 $\mu$g of endotoxin. Galanos *et al.* (1971) report that the LD50 of lipid A–BSA for mice is in excess of 1000 $\mu$g and in terms of " therapeutic index " it seems possible that the lipid A–BSA is superior to both endotoxin and dsRNA. However, further experiments with a large batch of

TABLE VIII.—*Effect of Endotoxin on Various Syngeneic Tumours Transplanted i.d.*

| Lymphoma | Antitumour effect† | Immunogenicity of tumour* |
|---|---|---|
| L5178Y (DBA/2) | + + + | + + + |
| L5178Y-M (DBA/2) | − | + − |
| TLX9 (C57Bl) | + − | + − |
| TLC5 (CBA) | + − | + |
| SL2 (DBA/2) | + + + | + + |
| Fibrosarcoma | | |
| FS1 (C57Bl) | + + | + + + |
| FS6 (C57Bl) | + + | + + + |
| FS9 (CBA) | − | + |
| FS11 (CBA) | − | + |

* Immunogenicity of the above lymphomata has been defined in terms of the number of cells rejected after immunization with irradiated cells (the details are given by Parr, 1972). In the case of the sarcomata the gradation is based on experiments in which immunization was by means of excision of the tumour. These animals then resisted 5 × $10^6$ FS6 or FS1 sarcoma cells but with the CBA fibrosarcomata FS9, FS11, 5 × $10^6$ cells grew into tumour in 7/10 such animals.

† −No effect.

\+ − A slowing of growth rate in some of the tumours.

\+ + Some temporary regressions—no complete cures.

\+ + + Complete cures in > 50% of animals.

TABLE IX.—*Effect of Different Macromolecules on the L5178Y Lymphoma Growing i.d.*

| Substance | Dose/mouse injected i.p. day 7 after transplant | Necrosis | Effect on tumour growth*: None | Slowing | Temporary regression | Permanent regression |
|---|---|---|---|---|---|---|
| dsRNA | 100 μg (5 times) twice weekly | + + | 4/15 | 5/15 | 1/15 | 5/15 |
| dsRNA treated with RNAase | 100 μg (5 times) twice weekly | − | 5/5 | 0/5 | 0/5 | 0/5 |
| Poly I Poly C | 100 μg (5 times) twice weekly | + + | 2/6 | 2/6 | 0/6 | 1/6 |
| Endotoxin | 10 μg | + + + | 0/15 | 0/15 | 2/15 | 13/15 |
| Glycolipid | 50 μg | + + | 5/5 | 0/5 | 0/5 | 0/5 |
| | 100 μg | | 1/9 | 4/9 | 1/9 | 3/9 |
| Lipid A solubilized with BSA | 50 μg | + + + | 4/5 | 1/5 | 0/5 | 0/5 |
| | 250 μg | | 0/11 | 1/11 | 0/11 | 10/11 |
| Lipid A solubilized with pyridine | 250 μg | + + + | 0/9 | 4/9 | 0/9 | 5/9 |
| Lipid A solubilized with alkali | 250 μg | + + | 3/9 | 3/9 | 0/9 | 3/9 |

* Defined in Table II.

lipid A are needed to establish these quantitative aspects. From the point of view of mechanisms it is clear, however, that the polysaccharide components of endotoxin are not required for the anti-tumour action of endotoxin. Westphal and his colleagues (1954) had previously shown that several other biological activities of endotoxin were also closely mimicked by lipid A when suitably solubilized.

### C. *Mechanism of action*

1. *Failure to detect a direct cytotoxic action*

Alexander and Evans (1970) had shown that both lymphoma and sarcoma cells grow normally *in vitro* in the presence of 50 μg of dsRNA or endotoxin. However, at very much lower concentrations these macromolecules " activate " macrophages and render them cytotoxic to lymphoma and sarcoma cells. An apparent cytotoxic action of these agents can be sometimes observed in cultures of sarcoma cells established from cells obtained directly from a sarcoma. This effect, however, is mediated by macrophages. Sarcomata in mice and rats contain macrophages and sometimes up to 40% of the cells derived by enzyme digestion of a sarcoma are macrophages (Evans, 1972). Consequently, primary cultures established from the cells of a sarcoma can constitute a mixture of macrophages and sarcoma cells; dsRNA and endotoxin act on the macrophage component which then inhibits the growth of sarcoma cells. If the macrophages are removed from a culture of sarcoma cells then dsRNA and endotoxin at doses of 50 μg/ml fail to effect the growth of the sarcoma cells *in vitro*.

We decided to test whether endotoxin-treated tumour cells grew normally *in vivo* and accordingly $10^6$ L5178Y lymphoma or $10^6$ FS6 sarcoma cells prepared by trypsin treatment of the respective tumours were injected i.d. in 0·1 ml of tissue culture fluid together with 5 μg of endotoxin. The rate of tumour growth was quite normal and the endotoxin was without effect (see Table X). Cells teased mechanically from a sarcoma and injected immediately i.d. into mice were occasionally prevented from growing if 5 μg of endotoxin were added to the cells before injection. It was thought that this occasional inhibition might be due to an increase in the numbers of macrophages present in the cell suspensions prepared from the tumours. To test this, $4 \times 10^6$ macrophages from the peritoneal cavity of normal C57Bl mice were added to a cell suspension of FS6 prepared enzymically and the mixture injected i.d. into mice. This mixture of cells gave rise to normal tumours; however, if 5 μg of endotoxin was added to the mixtures of macrophages and sarcoma cells then there was marked interference with tumour growth (see Table X).

2. *Vascular effects of endotoxin*

Shwartzman (1937) and Andervont (1936) attributed the effect of endotoxin on established tumours to a haemorrhagic reaction. The changes produced by endotoxin in tumours are in many respects similar to a local Shwartzman reaction. In the latter, necrosis is produced by systemic administration of endotoxin fol-

TABLE X.—*Effect of* in vitro *Addition of Endotoxin*—in vivo *Growth Assay*

| | Effect on tumour growth* | | | |
|---|---|---|---|---|
| Cell mixture injected i.d. | None | Slowing | Temporary regression | Permanent regression |
| Lymphoma $2 \times 10^6$ cells + 5 μg endotoxin | 5/5 | 0/5 | 0/5 | 0/5 |
| Trypsinized suspension of FS6 $10^5$ cells + 5 μg endotoxin | 5/5 | 0/5 | 0/5 | 0/5 |
| Mechanical suspension of FS6 $10^5$ cells + 5 μg endotoxin | 10/15 | 0/15 | 2/15 | 3/15 |
| Trypsinized suspension of FS6 $10^5$ cells + $4 \times 10^6$ peritoneal macrophages | 5/5 | 0/5 | 0/5 | 0/5 |
| Trypsinized suspension of FS6 $10^5$ cells + 5 μg endotoxin + $4 \times 10^6$ peritoneal macrophages | 0/10 | 0/10 | 4/10 | 2/10 4/10† |

* Defined in Table II.

†No growth of tumour.

lowing an initial primary dose given locally. The tumour vasculature without prior exposure to endotoxin seems to respond to endotoxin like the blood vessels in skin after initial sensitization. Since the local Shwartzman reaction can be inhibited by administration of heparin (Good and Thomas, 1953), the effect of full heparinization on the antitumour action of endotoxin was studied with both L5178Y lymphoma and the FS6 sarcoma.

Twenty mice were heparinized on Day 7 after inoculation i.d. of $10^6$ L5178Y cells. Endotoxin (10 $\mu$g per mouse) was injected into 10 of these animals 4 hours after the first dose of heparin. Injections ofheparin (3 per 24 hours) were continued for a total period of 4 days. The haemorrhagic necrotic action was rather slower to appear in the tumours of the heparinized animals but the usual scabs had formed over the tumours before the period of heparinization was over. Some tumour regression was observed in the heparinized mice but the effect was greatly reduced when compared with that seen in normal mice (Table XI). In a similar experiment, animals carrying FS6 fibrosarcomata were heparinized and then treated with endotoxin. Tumour necrosis was reduced and the inhibition of tumour growth was only slight in the heparinized group (Table XI).

### 3. *Effect of an α-adrenergic antagonist " dibenyline "*

Some of the toxic manifestations of endotoxin are attributable to the release of catecholamines and consequent slowing of blood flow (Levy and Blattberg, 1964). This can be prevented by α-adrenergic blockade and dibenyline is used clinically to protect against the irreversible shock syndrome of endotoxin poisoning (Levy, North and Wells, 1954; Eckenhoff and Cooperman, 1965). To test if the slowing of blood flow due to the constriction of arteriolar vessels plays a part in the antitumour action of endotoxin, DBA/2 mice bearing intradermal L5178Y lymphoma were injected i.p. with 100 $\mu$g of dibenyline 2 hours before giving 10 $\mu$g of endotoxin i.p. This treatment did not reduce the haemorrhagic necrosis following endotoxin but a slightly decreased antitumour effect was recorded (Table XII). In a further experiment where the dose of dibenyline was increased from 100 $\mu$g to 500 $\mu$g/mouse, the signs of haemorrhagic necrosis were slower to appear in the tumours of the dibenyline–endotoxin group of animals, but tumour regression still occurred.

However, dibenyline protected powerfully against the toxic effects of endotoxin and made it possible to use much higher doses of the latter. The possibility that higher doses of endotoxin in mice protected by dibenyline may cause tumours to regress which had failed to respond to 10 $\mu$g of endotoxin was investigated with the fibrosarcoma FS9. CBA mice carry-9-day-old FS9 fibrosarcomata were injected

TABLE XI.—*Effect of Heparin on Antitumour Action of Endotoxin*

| | | | Effect on tumour growth* | | | |
|---|---|---|---|---|---|---|
| Tumour | Treatment | Average diameter of tumour (mm) on day of treatment ( ) | None | Slowing | Temporary regression | Permanent regression |
| L5178Y† | Endotoxin 10 $\mu$g | 7·5 (7) | 0/10 | 0/10 | 1/10 | 9/10 |
| | Heparin before and after 10 $\mu$g endotoxin‖ | 8·0 (7) | 0/9 | 4/9 | 3/9 | 2/9 |
| FS6§ | Endotoxin 10 $\mu$g | 6·0 (10) | 1/10 | 3/10 | 5/10 | 1/10 |
| | Heparin before and after 10 $\mu$g endotoxin‖ | 7·0 (10) | 1/8 | 6/8 | 1/8 | 0/8 |

* Defined in Table II.

† 2 × $10^6$ L5178Y cells were injected i.d.

§ 5 × $10^5$ FS6 cells were injected i.d.

‖ 100 $\mu$g heparin 3 × per 24 hours s.c.

TABLE XII.—*Attempts to Block Antitumour Effects of Endotoxin with Dibenyline*

| Tumour | Treatment | Average diameter of tumour (mm) on day of treatment ( ) | Degree of haemorrhagic necrosis | Effect on tumour growth*: None | Slowing | Temporary regression | Permanent regression |
|---|---|---|---|---|---|---|---|
| L5178Y‖ | Endotoxin (10 μg) | 7·5 (7) | + + + + | 0/10 | 0/10 | 1/10 | 9/10 |
| | †Dibenyline 2 hours before 10 μg endotoxin | 7·5 (7) | + + + + | 2/10 | 2/10 | 0/10 | 6/10 |
| FS9⊥ | Endotoxin (10 μg) | 8·5 (10) | + | 14/15 | 3/15 | 0/15 | 0/15 |
| | Dibenyline 2 hours before 100 μg§ endotoxin | 7·5 (10) | + + | 0/5 | 2/5 | 2/5 | 1/5 |

* Defined in Table II.

† 100 μg i.p. 2 hours before endotoxin also injected i.p.

§ 100 μg endotoxin caused 100% mortality in CBA mice.

‖ 2 × $10^6$ cells injected i.d.

⊥ $10^5$ cells injected i.d.

with 100 μg of endotoxin 2 hours after i.p injection of 100 μg of dibenyline. Table XII shows that this tumour responded to the higher dose of endotoxin which had been made possible by protection with dibenyline. We conclude that the biological effects of endotoxin, which are protected against by dibenyline, do not play a significant role in causing tumour regression and that in combination with dibenyline endotoxin can be more effectively employed. Dibenyline, however, failed to protect against the toxic effects of dsRNA and did not make it possible to use a higher dose of this compound.

4. *Effects of immunosuppression*

In order to investigate the involvement of immune factors in the antitumour effects of dsRNA and endotoxin, the immune response of the host to its own tumour was suppressed by either whole body irradiation with x-rays, by corticosteroids or by antilymphocyte serum.

As can be seen from Table XIII whole body irradiation totally abolished the antitumour action against the i.d. lymphoma by endotoxin and by dsRNA. In this series of experiments the dsRNA produced no permanent regression in the control group and there were only

TABLE XIII.—*Effect of Whole Body x-irradiation on Antitumour Action of dsRNA and Endotoxin*

| | L5178Y lymphoma: Endotoxin | | L5178Y lymphoma: dsRNA | | FS6 sarcoma endotoxin | |
|---|---|---|---|---|---|---|
| Effect on tumour growth* | Normal | x-irradiation | Normal | x-irradiation | Normal | x-irradiation |
| None | 0/15 | 15/15 | 2/5 | 5/5 | 0/5 | 1/5 |
| Slowing of tumour growth | 2/15 | 0/15 | 1/5 | 0/5 | 2/5 | 4/5 |
| Temporary regression | 0/15 | 0/15 | 2/5 | 0/5 | 2/5 | 0/5 |
| Permanent regression | 13/15 | 0/15 | 0/5 | 0/5 | 1/5 | 0/5 |

* Defined in Table II.
Whole body irradiation of 400 rad given 24 hours before tumour transplant. Endotoxin (10 μg) and dsRNA (100 μg 2× weekly) given i.p. Day 7 after transplant i.d. of $10^6$ tumour cells.

temporary regressions and a slowing of growth. In animals that had been irradiated dsRNA was totally without effect. The influence of whole body irradiation on the action of endotoxin against the fibrosarcoma was not so dramatic but there was a reduction of response. This series of experiments does not by itself prove that an intact immune mechanism is needed for the antitumour action of dsRNA and endotoxin to be manifested, since whole body irradiation causes widespread pathological changes and in particular induces leucocytopenia. However, two other treatments which are immunosuppressive, namely administration of ALS and of cortisone, both reduce the antitumour action of endotoxin (Table XIV). It is difficult to envisage an effect other than immunosuppression which is shared by whole body irradiation and treatment with ALS or cortisone, and while the hypothesis that an intact immune system is needed for endotoxin and dsRNA to cause regression of established tumours has not been unambiguously proven by these experiments, it is extremely likely. It is of interest that haemorrhagic necrosis still occurred in tumours of endotoxin-treated mice that had been exposed to whole body irradiation. Intradermal L5178Y tumours growing in mice subjected to 400 rad 24 hours before tumour transplant, were treated with 1 μg, 5 μg or 10 μg per mouse of endotoxin and the degree of haemorrhagic necrosis occurring in the tumours assessed in a "blind" study by two individuals. It was found that although haemorrhagic necrosis did not occur as quickly in the irradiated mice and did not, except in the group given 10 μg of endotoxin, reach quite the same intensity, it did still occur whereas the antitumour activity of endotoxin was completely absent in the irradiated mice (Table XIII).

### DISCUSSION

With regard to all of the toxic manifestations, including pyrexia, shock syndromes, enhancement of toxicity by lead acetate and temperature elevation, effect on complement and blood clotting systems and intravascular coagulation, as well as the activation of macrophages, dsRNA (both of viral origin and synthetic Poly I Poly C) cannot be distinguished qualitatively from endotoxin and, as far as the limited data warrant, from lipid A. In this paper we have demonstrated that this similarity in activities also applies to the antitumour actions. These substances appear to have two quite distinct effects on tumour growth (1) prophylactic when given several days before challenge and (2) therapeutic when given several days after tumour implantation. With

TABLE XIV.—*Effect of ALS and Cortisone on Antitumour Action of Endotoxin on Intradermal L5178Y*

| Treatment | Day of treatment after tumour transplant | Average diameter of tumour on day of treatment (mm) | Effect on tumour growth*: None | Slowing | Temporary regression | Permanent regression |
|---|---|---|---|---|---|---|
| None | | | 5/5 | 0/5 | 0/5 | 0/5 |
| Endotoxin† | 16 | 12 | 0/5 | 0/5 | 5/5 | 0/5 |
| ALS and endotoxin§ | 16 | 12 | 3/5 | 2/5 | 0/5 | 0/5 |
| Endotoxin | 7 | 6·9 | 0/5 | 0/5 | 1/5 | 4/5 |
| Cortisone and endotoxin‖ | 7 | 7·1 | 2/5 | 1/5 | 1/5 | 1/5 |

* Defined in Table II.
† 10 μg endotoxin injected i.p.
§ Injections of ALS given i.p. every 2 days beginning 2 days after tumour transplant.
‖ 1·25 μg prednisolone acetate injected s.c. 4 hours before endotoxin.

the tumours used in this study the prophylactic action is only observed against an i.p. challenge (see Table V) and not against an s.c. challenge and, moreover, for protection to be observed dsRNA or endotoxin must be given several days before challenge; they are without effect if given one day before the tumour. The requirements for therapy are diametrically opposite: s.c. and i.d. respond but ascites tumours do not. A response in s.c. and i.d. tumours is seen only if treatment is given several days after tumour transplant.

The protective activity correlates with the presence in the peritoneal cavity of activated macrophages (Alexander and Evans, 1971). Immediately after injection of dsRNA or endotoxin, macrophages seem to disappear from the peritoneal cavity and only very small numbers can be recovered from peritoneal washings. By the fourth day numbers increase again and by the seventh day macrophages can be recovered in normal yields. These, however, are activated in that they kill tumour cells *in vitro*. The hypothesis that the protective action seen only in the peritoneal cavity and not at subcutaneous sites is due to activated macrophages, is strengthened by recent studies of Hibbs, Lambert and Remington (1971) who find that mice chronically infected with microorganisms growing intracellularly are more resistant to tumour challenge and that their peritoneal macrophages are " activated ".

The therapeutic effect of dsRNA and endotoxin is more complex and the data presented indicate that several factors contribute. Clearly these agents do not act directly on the tumour cells since their growth both *in vitro* and *in vivo* is unaffected following exposure to dsRNA and endotoxin. Damage of blood vessels in the tumour would appear to be important but not by itself sufficient to cause the regressions. The reduction of the antitumour activity by heparin establishes the key role of intravascular coagulation in this process and provides an immediate explanation why only established solid tumours (*i.e.*, those that have become fully vascularized) respond and why ascites tumours are quite unaffected. On the other hand, dsRNA and endotoxin still induce haemorrhage in the tumours of animals that have been exposed to 400 rad of whole body irradiation and in tumours of animals that have received ALS or cortisone, yet these pre-treatments abolish the antitumour action of endotoxin and of dsRNA. Also, endotoxin and dsRNA induce haemorrhagic necrosis in some tumours without halting their growth.

That an immune reaction of the host directed against the tumour contributes to the tumour regression induced by dsRNA and endotoxin is indicated by the fact that only strongly antigenic tumours respond to these treatments. Moreover, in animals that have been deliberately immunosuppressed by ALS or whole body irradiation, tumour regressions are not induced. Admittedly these pre-treatments also affect the blood clotting system, but since haemorrhagic necrosis still occurs it seems more likely that it is immunosuppression, and not some other pathology induced by both x-rays and ALS, which counteracts the antitumour activity of dsRNA and endotoxin. The failure of dibenyline to block the antitumour action indicates that this effect is not mediated *via* the action of endotoxin on the adrenergic nervous system.

The apparent need for both haemorrhagic necrosis and an active immune response suggests that the effect of dsRNA and of endotoxin on tumours is due to a breakdown of the vascular structure which allows the various immunological effector mechanisms of the host to gain access to the tumour. Physical inaccessibility of cells in a solid tumour is one reason for the relative immunotherapeutic ineffectiveness of antisera and the same may well apply to some of the cell-mediated components of the immune response. Vascular damage alone can only cause a temporary slowing of tumour growth, but when this occurs in a host which is reacting to an antigenic tumour, then the combined response may result in the regressions that are observed.

In addition to haemorrhagic necrosis and host immunity, activation of macrophages may be another and parallel mechanism in the antitumour reaction. Evans (1972) has shown that tumours can contain many macrophages, thus in the FS6 sarcoma which responds to dsRNA and endotoxin at least 40% of the cells are macrophages while the FS9—a tumour which does not usually respond—contains only 20% macrophages.

Thus it is likely there are several factors contributing to the tumour regression brought about by endotoxin and dsRNA and for the various tumour systems that respond, these factors may contribute in differing degrees depending on the nature of the tumour and the route of administration. The antitumour effects of dsRNA and endotoxin in L5178Y lymphoma and fibrosarcoma FS6 differed in two respects. Firstly, complete abolition of the dsRNA or endotoxin-induced tumour regressions by immunosuppression occurred only with the lymphoma, whereas with the fibrosarcoma the antitumour effects were reduced by x-irradiation but not completely eliminated. This latter result is more in agreement with experiments of Fischer *et al.* (1972) who utilized a fibrosarcoma and a polyoma for their work. Secondly, the effect of dsRNA injected directly into the tumour was not increased in the case of the lymphoma by repeated treatment whereas the opposite was true of FS6 fibrosarcoma (Table VI). These differences might be explained in terms of the varying contributions of the two processes capable of causing tumour regression, for example (1) vascular damage with the ensuing release of immune factors and (2) macrophage activation. The first mechanism would be sensitive to immunosuppression whereas there is evidence that macrophage activation would not. *In vitro* experiments by den Otter (unpublished) show that macrophages that have been subjected to x-irradiation can still be activated by addition of dsRNA or endotoxin. The low macrophage content of intradermal L5178Y lymphomata (personal observation) compared to the 40% present in FS6 fibrosarcomata (Evans, 1972) could be taken as further evidence that vascular damage rather than macrophage activation plays a key role in the dsRNA–endotoxin induced tumour necrosis of lymphoma tissue. On the other hand, macrophage activation could be an important component of tumour regressions induced in the fibrosarcomata. The observed increase in tumour regression following large doses of endotoxin to mice carrying the less antigenic fibrosarcoma FS9 might be explained in terms of activation of increased numbers of macrophages. On the basis of this hypothesis one could predict that where macrophages form part of the structure of the tumour and if the drug can be supplied in sufficient concentration locally to the tumour, then therapy would be successful. Preliminary results suggest that treatment of animals carrying lung tumour (FS6 fibrosarcoma cells injected i.v.) with large doses of endotoxin can prolong survival times. The available data are consistent with the hypothesis that the contribution of macrophage activation to the antitumour effect will be greatest for tumours rich in macrophages and when dsRNA and endotoxin are given directly into the tumour. When given systemically the mechanism requiring vascular damage coupled with an active immune reaction by the host may predominate.

The finding that the polysaccharide-free endotoxin derivative lipid A has the structure shown in Fig. 5 (Gmeiner *et al.*, 1969), allows some speculation concerning a possible molecular basis for the similarity in action of dsRNA and endotoxin. It is not inconceivable that both may bind to a common receptor on cells which relies for its specificity on a combination of recurring phosphate groups, present in both macromolecules, and of a hydroxyl group provided by the myristic acid in lipid A and by ribose in dsRNA.

FIG. 5.—Structure of a lipid A unit with a KDO oligosaccharide. FS = long chain fatty acids. HM = $\beta$-hydroxymyristic acid. Published Gmeiner *et al.* (1969).

We wish to thank Dr R. L. Carter for preparation and interpretation of the histological data, Professor O. Westphal for supplying the endotoxin derivatives, Dr D. A. L. Davis for the Shigella endotoxin and Beecham's Research Laboratories Ltd for the dsRNA.

This work was supported by grants made to the Chester Beatty Research Institute by the Medical Research Council and the Cancer Research Campaign and by a grant from the Napier Trust.

### REFERENCES

ALEXANDER, P. & EVANS, R. (1971) Endotoxin and Double Stranded RNA render Macrophages cytotoxic. *Nature, New Biol.*, **232**, 76.

ANDERVONT, H. B. (1936) The Reaction of Mice and of various Mouse Tumours to the Injection of Bacterial Products. *Am. J. Cancer*, **27**, 77.

BUSCH, –. (1866) Verhandlungen ärztlicher Gesellschaften. *Klin. Wschr.*, **3**, 245.

DRÖGE, W., LEHMANN, V., LÜDERITZ, O. & WESTPHAL, O. (1970) Structural Investigations on the 2-keto-3-deoxyoctonate Region of Lipopolysaccharides. *Eur. J. Biochem.*, **14**, 175.

ECKENHOFF, J. E., COOPERMAN, L. H. (1965) The Clinical Application of Phenoxybenzamine in Shock and Vasoconstrictive states. *Surgery, Gynec. Obstet.*, **121**, 483.

EVANS, R. (1972) Macrophages in Syngeneic Animal Tumours. *Transplantation*, **14**, 468.

FISCHER, J. C., COOPERBAND, S. R. & MANNICK, J. A. (1972) The Effect of Polyinosinic–polycytidylic acid on the Immune Response of Mice to Antigenically Distinct Tumours. *Cancer Res.*, **32**, 889.

GALANOS, C., RIETSCHEL, E. T., LÜDERITZ, O. & WESTPHAL, O. (1971) Interaction of Lipopolysaccharides and Lipid A with Complement. *Eur. J. Biochem.*, **19**, 143.

GMEINER, J., LÜDERITZ, O. & WESTPHAL, O. (1969) Biochemical Studies on Lipopolysaccharides of Salmonella R mutants. 6. Investigations on the structure of the Lipid A Component. *Eur. J. Biochem.*, **7**, 370.

GOOD, R. A. & THOMAS, L. (1953) Studies on the Generalized Shwartzman Reaction. IV. Prevention of the Local and Generalized Shwartzman Reactions with Heparin. *J. exp. Med.*, **97**, 871.

HALPERN, B. N., BIOZZI, G., STIFFEL, C. & MOULTON, D. (1959) Corrélation entre l'activité phagocytaire du système réticulo-endothélial et la production d'anticorps antibactériens. *C.r. Soc. Biol.*, **153**, 919.

HALPERN, B. N., BIOZZI, G., STIFFEL, C. & MOULTON, D. (1966) Inhibition of Tumour Growth by Administration of killed Corynebacterium parvum. *Nature, Lond.*, **212**, 853.

HIBBS, J. B., JR., LAMBERT, L. H., JR. & REMINGTON, J. S. (1971) Tumour Resistance conferred by Intracellular Protozoa. *J. clin. Invest.*, **50**, 45a.

HIBBS, J. B., JR., LAMBERT, L. H., JR. & REMINGTON, J. S. (1972) Possible Role of Macrophage mediated Nonspecific Cytotoxicity in Tumour Resistance. *Nature, New Biol.*, **235**, 48.

HILLEMAN, M. R. (1970) Double-stranded RNAs (Poly I : C) in the Prevention of Viral Infections. *Archs intern. Med.*, **126**, 109.

LEVY, E. Z., NORTH, W. C. & WELLS, J. A. (1954) Modification of Traumatic Shock by Adrenergic Blocking Agents. *J. Pharmac. exp. Ther.*, **112**, 151.

LEVY, H. B., LAW, L. W. & RABSON, A. S. (1969) Inhibition of Tumour Growth by Polyinosinic-polycytidylic acid. *Proc. natn. Acad. Sci.*, **62**, 357.

LEVY, H. B. (1970) Interferon and Interferon inducers in the Treatment of Malignancies. *Archs intern. Med.*, **126**, 78.

LEVY, M. N. & BLATTBERG, B. (1964) In *Shock*. Ed. S. G. Hershey. Boston: Little, Brown and Co.

LÜDERITZ, O. (1970) Recent Results on the Biochemistry of the Cell Wall. Lipopolysaccharides of Salmonella Bacteria. *Angew. Chem.*, **9**, 649.

MATHÉ, G., POUILLART, P. & LAPEYRAQUE, F. (1969) Active Immunotherapy of L1210 Leukaemia Applied after the Graft of Tumour Cells. *Br. J. Cancer*, **23**, 814.

NAUTS, H. C., FOWLER, G. A. & BOGATKO, F. H. (1953) A Review of the Influence of Bacterial Infection and of Bacterial Products (Coley's Toxins) on Malignant Tumours in Man. *Acta med. scand.*, **45**, Suppl. No. 276.

OLD, L. J., BENACERRAF, B., CLARKE, D. A., CARSWELL, E. A. & STOCKERT, E. (1961) The Role of the Reticulo-endothelial System in the Host Reaction to Neoplasia. *Cancer Res.*, **21**, 1281.

PARR, I. (1972) Response of Syngeneic Murine Lymphomata to Immunotherapy in Relation to Antigenicity of the Tumour. *Br. J. Cancer*, **26**, 174.

PLANTEROSE, D. N., BIRCH, P. J., PILCH, D. J. F. & SHARPE, T. J. (1970) Antiviral Activity of dsRNA and virus-like Particles from Penicillium Stoloniferum. *Nature, Lond.*, **227**, 504.

PILCH. D. J. F. & PLANTEROSE, D. N. (1971) Effects on Friend Disease of double-stranded RNA of Fungal Origin. *J. gen. Virol.*, **10**, 155.

SHWARTZMAN, G. (1937) *Phenomenon of Local Tissue Reactivity and its Immunological, Pathological and Clinical Significance.* New York: P. B. Hoeber Inc. p. 461.

SUTTHIWAN, P., SHORTER, R. G., HALLENBECK, G. A. & ELVEBACK, L. R. (1969) Comparison of Rabbit Anti-mouse thymus sera Produced after Different Numbers of Injections of Lymphoid Cells. *Transplantation*, **8**, 249.

WESTPHAL, O. & LÜDERITZ, O. (1954) Chemische Erforschung von Lipopolysacchariden gramnegativer Bakterien. *Angew. Chem.*, **66**, 407.